# Astrocytic gamma-aminobutyric acid dysregulation as a therapeutic target for posttraumatic stress disorder

**DOI:** 10.1038/s41392-025-02317-5

**Published:** 2025-07-28

**Authors:** Sujung Yoon, Woojin Won, Suji Lee, Kayoung Han, Eunji Ha, Juheon Lee, Seung Jae Hyeon, Yoonji Joo, Haejin Hong, Hyangwon Lee, Yumi Song, Ki Duk Park, Bertrand R. Huber, Junghee Lee, Richard A. E. Edden, Minah Suh, Hoon Ryu, C. Justin Lee, In Kyoon Lyoo

**Affiliations:** 1 Ewha Brain Institute, Ewha Womans University, Seoul, South Korea; 2 Department of Brain and Cognitive Sciences, Ewha Womans University, Seoul, South Korea; 3 Center for Cognition and Sociality, Institute for Basic Science (IBS), Daejeon, South Korea; 4 Division of Psychology and Cognitive Science, Seoul Women’s University, Seoul, South Korea; 5 Biomedical Institute for Convergence at SKKU (BICS), Sungkyunkwan University, Suwon, South Korea; 6 Center for Neuroscience Imaging Research, Institute for Basic Science (IBS), Suwon, South Korea; 7 Department of Biomedical Engineering, Sungkyunkwan University, Suwon, South Korea; 8 Department of Intelligent Precision Healthcare Convergence (IPHC), Sungkyunkwan University, Suwon, South Korea; 9 Center for Brain Disorders, Brain Science Institute, Korea Institute of Science and Technology (KIST), Seoul, South Korea; 10 Division of Bio-Med Science & Technology, KIST School, Korea University of Science and Technology, Seoul, South Korea; 11 VA Boston Healthcare System, U.S. Department of Veteran Affairs, Boston, MA USA; 12 Boston University Alzheimer’s Disease Research Center and Department of Neurology, Boston University Chobanian & Avedisian School of Medicine, Boston, MA USA; 13 The Russell H. Morgan Department of Radiology and Radiological Science, Johns Hopkins University School of Medicine, Baltimore, MD USA; 14 Graduate School of Pharmaceutical Sciences, Ewha Womans University, Seoul, South Korea

**Keywords:** Neurological disorders, Cellular neuroscience, Molecular neuroscience, Target validation

## Abstract

Post-traumatic stress disorder (PTSD) remains a debilitating psychiatric condition with limited pharmacological treatment options. Identifying novel therapeutic targets is critical for addressing its unmet clinical needs. Through our comprehensive human clinical research, including both cross-sectional and longitudinal studies, we revealed a compelling link between dysregulated prefrontal gamma-aminobutyric acid (GABA) levels and PTSD symptoms. Notably, elevated prefrontal GABA levels in PTSD patients are associated with impaired cerebral blood flow (CBF) and symptom severity, normalizing with recovery, highlighting GABA dysregulation as a key mechanism in the disorder. Postmortem and PTSD-like mouse models implicated monoamine oxidase B (MAOB)-dependent astrocytic GABA as a primary driver of this imbalance, exacerbating deficit in fear extinction retrieval. Genetic and pharmacological inhibition of MAOB effectively restored astrocytic GABA and improved fear extinction retrieval in PTSD-like mouse models. Specifically, KDS2010, a recently developed highly selective and reversible MAOB inhibitor, not only restored astrocytic GABA homeostasis but also rescued CBF deficits and reduced tonic GABA and astrogliosis in the prefrontal cortex. Moreover, KDS2010 successfully advanced through Phase 1 clinical trials, showing a favorable safety profile and paving the way for Phase 2 trials to evaluate its therapeutic potential in PTSD. Our findings highlight the pivotal role of astrocytic GABA in PTSD pathophysiology and establish MAOB inhibition as a mechanistically targeted approach to alleviate symptoms. By bridging human and animal studies with translational clinical trials, this work positions KDS2010 as a promising first-in-class therapy, offering a novel paradigm for PTSD treatment.

## Introduction

Post-traumatic stress disorder (PTSD) is a debilitating condition characterized by a range of symptoms, including intrusive thoughts, avoidance of trauma-related stimuli, hyperarousal, and arising from the inability to regulate emotional responses toward traumatic memories properly.^1^ Despite the profound impact of PTSD on personal, social, and health outcomes,^1^ current pharmacological treatments are limited, with only two serotonin-receptor-targeting drugs officially approved.^2^ However, even with these treatments, only 20–30% of patients achieve complete remission,^3^ highlighting a critical need for novel mechanism-based therapeutic strategies to address symptoms of PTSD.

The prefrontal cortex (PFC) plays a pivotal role in regulating fear extinction, a critical process for trauma recovery. Specifically, the infralimbic (IL) and prelimbic (PL) regions of the PFC modulate fear extinction, with IL activation shown to facilitate this process in animal models.^4–6^ Given that individuals with PTSD exhibit impaired fear extinction, the inhibitory control of PFC is associated with both the development of PTSD and the potential for long-term recovery from it.^7,8^ Furthermore, gamma-aminobutyric acid (GABA) signaling in the PFC and amygdala has been implicated in fear acquisition and extinction processes.^9,10^ Dysregulated GABAergic activity in the PFC is associated with impaired fear extinction,^11,12^ a hallmark of PTSD symptoms. Although GABA level changes may vary across brain regions, participant characteristics, or technical differences in MRS protocols, emerging evidence from proton magnetic resonance spectroscopy (^1^H-MRS) studies suggests potential alteration in prefrontal GABA levels during acute stress and in individuals with PTSD.^13,14^ Additionally, cortical GABAergic dysfunction has been reported in a brain positron emission tomography study of PTSD.^15^ Collectively, these findings suggest that GABA dysregulation contributes to impaired cortical connectivity and fear extinction deficits,^10,14^ suggesting GABA is a potential therapeutic target for PTSD intervention.

Our recent studies highlight the pivotal role of astrocyte-derived tonic GABA in modulating neural activity, including sensory information processing, memory formation, and plasticity.^16^ Astrocytes synthesize tonic GABA via monoamine oxidase B (MAOB) and degrade it through 4-aminobutyrate aminotransferase (ABAT), which fine-tune neural activity under both physiological and pathological conditions.^17,18^ Moreover, putrescine, a polyamine pre-substrate for MAOB, links polyamine metabolism to astrocytic GABA production.^19^ Dysregulated putrescine metabolism may enhance MAOB activity, increasing GABA synthesis and exacerbating tonic inhibition under pathological conditions. Under pathological conditions, reactive astrocytes undergo hypertrophy, upregulate MAOB, and produce excessive GABA, leading to abnormal tonic inhibition of synapses.^17,20^ This mechanism has been implicated in neuropsychiatric disorders such as Alzheimer’s disease, Parkinson’s disease, epilepsy, and anxiety-like behaviors,^16,21,22^ suggesting a broader role for astrocytic GABA dysregulation in brain dysfunction. Given the elevated MAOB expression observed in psychiatric disorders and its potential to drive GABA dysregulation, we hypothesize that aberrant astrocytic GABA disrupts inhibitory balance and impairs fear extinction in PTSD.

**Fig. 1:**
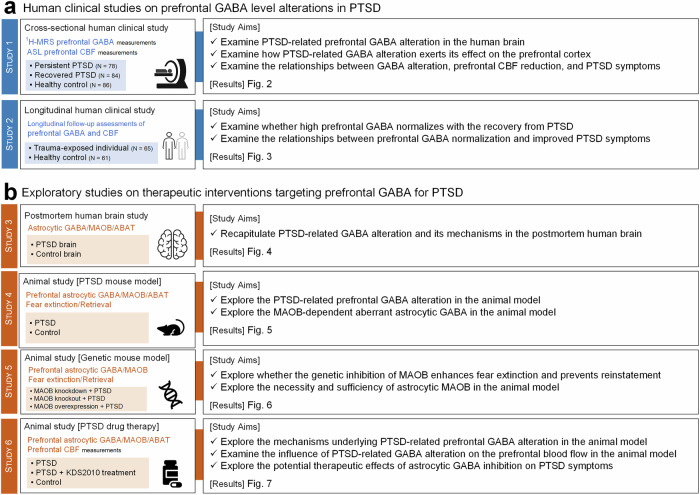
Overview of study design and aims. **a** The first part of this research consisted of two comprehensive human clinical studies aimed at examining alterations in prefrontal GABA levels and their clinical implications in PTSD. Cross-sectional clinical study analyzed a cohort of 248 participants, divided into groups of persistent PTSD (*N* = 78), recovered PTSD (*N* = 84), and healthy control (*N* = 86), to investigate prefrontal GABA alterations in PTSD and their impact on CBF and clinical symptoms. Longitudinal clinical study involved 126 participants, including a trauma-exposed group (*N* = 65) and a healthy control group (*N* = 61), to study the reversibility of prefrontal GABA alterations as PTSD symptoms improved over time. **b** The second part of this research explored the therapeutic potential and underlying cellular mechanisms of interventions targeting prefrontal GABA inhibition for PTSD treatment. Postmortem human brain study focused on elucidating the cellular mechanisms underlying prefrontal GABA alterations in PTSD through the analysis of postmortem human brain tissues. PTSD mouse model study utilized a PTSD-like animal model to evaluate the aberrant astrocytic GABA homeostasis, confirming the consistency with humans. Genetic mouse model study investigated the potential benefits of genetically inhibiting MAOB to facilitate fear extinction in PTSD mouse models, suggesting implications for therapeutic strategies. PTSD mouse model study with KDS2010 treatment utilized a PTSD-like mouse model to evaluate the therapeutic efficacy of KDS2010, a reversible, selective MAOB inhibitor, in treating PTSD by modulating prefrontal GABA synthesis pathways. PTSD post-traumatic stress disorder, GABA gamma-aminobutyric acid, ^1^H-MRS proton magnetic resonance spectroscopy, ASL arterial spin labeling, CBF cerebral blood flow, MAOB monoamine oxidase B, ABAT 4-aminobutyrate aminotransferase

To test this hypothesis, we adopted an integrative approach combining large-scale human clinical investigations, postmortem brain analyses, and PTSD-like mouse models. Our cross-sectional and longitudinal study revealed altered prefrontal GABA levels and disrupted cerebral blood flow (CBF) in PTSD, which correlated with symptom severity and recovery trajectories. Postmortem analyses identified astrocytic GABA dysregulation, implicating MAOB and ABAT as key regulators. Complimentary preclinical models confirmed these findings and demonstrated that genetic and pharmacological inhibition of MAOB effectively restored GABA balance and enhanced fear extinction retrieval, highlighting its therapeutic potential for PTSD treatments (Fig. 1).

## Results

### Prefrontal GABA alterations influence PTSD symptom severity through modulation of cerebral blood flow (cross-sectional clinical study)

The study subjects of cross-sectional clinical study consisted of three groups: individuals with PTSD (*N* = 78, referred to as the persistent PTSD group), individuals who had recovered from PTSD (*N* = 84, referred to as the recovered PTSD group), and healthy individuals without trauma exposure (*N* = 86, referred to as the healthy control group). This study aimed to determine whether prefrontal GABA levels are altered in chronic PTSD by comparing the persistent PTSD group to both the recovered PTSD group and the healthy control group.

Demographically, there were no significant differences among the groups in terms of age, sex, and handedness. Additionally, both the persistent and recovered PTSD groups displayed similar characteristics in terms of age at trauma, time elapsed since the trauma, and type of trauma experienced. These matched characteristics minimize confounding variables, suggesting that differences in GABA and CBF likely reflect PTSD pathophysiology rather than demographic or trauma-specific factors. As anticipated, individuals with persistent PTSD exhibited higher scores for PTSD symptoms compared to those in the recovered PTSD group, as detailed in Supplementary Table 1.

**Fig. 2:**
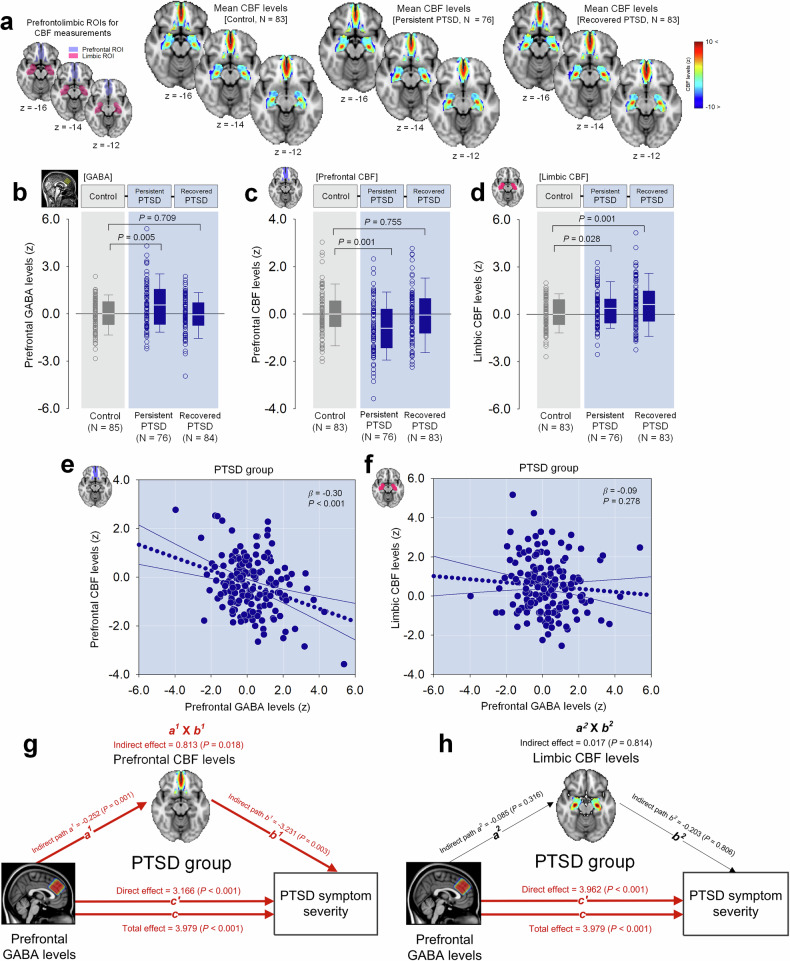
[Cross-sectional clinical study] PTSD-related prefrontal GABA alteration, CBF, and symptom severity. **a** Prefrontal and limbic ROIs for CBF measurement are depicted on an axial T1-weighted image. The right panels display color-coded mean CBF maps for the healthy control, persistent PTSD, and recovered PTSD groups. **b** Prefrontal GABA levels, assessed using ^1^H-MRS, were elevated in the persistent PTSD group compared to healthy controls, while levels in the recovered PTSD group were similar to those of controls. **c** The persistent PTSD group exhibited reduced prefrontal CBF relative to healthy controls, whereas the recovered PTSD group showed prefrontal CBF comparable to the control group. **d** Both persistent and recovered PTSD groups showed higher limbic CBF levels compared to the healthy control group. **e** In the combined PTSD group (persistent and recovered), higher prefrontal GABA levels were associated with lower prefrontal CBF. **f** No significant association was found between prefrontal GABA and limbic CBF levels in the PTSD group. **g** Mediation analysis indicated that the link between elevated prefrontal GABA levels and greater PTSD symptom severity could potentially be mediated by reduced prefrontal CBF. **h** However, no mediation effect was observed for limbic CBF. Error bars in the graphs indicate standard errors of the mean. PTSD post-traumatic stress disorder, ROI region-of-interest, VOI voxel-of-interest, CBF normalized resting-state cerebral blood flow, GABA gamma-aminobutyric acid, ^1^H-MRS proton magnetic resonance spectroscopy, MRI magnetic resonance imaging

This study measured prefrontal GABA levels and normalized resting-state CBF within the prefrontal area using ^1^H-MRS and arterial spin labeling (ASL) perfusion magnetic resonance imaging (MRI), respectively. These measurements were then compared across the three groups (Fig. 2a and Supplementary Fig. 1).

We observed that prefrontal GABA levels in the persistent PTSD group were significantly higher than those in the control group. This difference was not observed in the recovered PTSD group, where GABA levels were comparable to the control group (Fig. 2b). Additionally, the persistent PTSD group exhibited lower CBF in the prefrontal area and higher CBF in the limbic area than the healthy control group (Fig. 2c, d). Conversely, prefrontal CBF levels in the recovered PTSD group were similar to those in the healthy control group, although limbic CBF levels remained higher compared to the healthy controls (Fig. 2c, d). These findings suggest that alterations in prefrontal GABA levels and CBF levels may reflect neurobiological differences associated with PTSD and its recovery. Detailed statistical values are presented in Supplementary Table 3.

Subsequently, we investigated the correlation between prefrontal GABA levels and CBF levels in the prefrontal area among participants in both the persistent and recovered PTSD groups. Elevated prefrontal GABA levels were significantly associated with reduced prefrontal CBF (Fig. 2e). This inverse relationship highlights a potential neurovascular mechanism by which excessive GABAergic inhibition may reduce metabolic activity and impair cortical connectivity, further exacerbating PTSD symptoms. However, there was no significant correlation between prefrontal GABA levels and limbic CBF (Fig. 2f). Detailed statistical values are presented in Supplementary Table 4.

The robustness of our regression estimates against the presence of influential observations was assessed by conducting sensitivity analyses. Specifically, we re-estimated the models after excluding observations with Cook’s distance values exceeding the conventional threshold of 4/N.^23,24^ The results remained consistent even after removing these potentially influential data points. Specifically, after removing 12 potentially influential observations, prefrontal GABA levels were significantly elevated in the persistent group compared to the control group (β = 0.149, *P* = 0.046). There was no significant difference in prefrontal GABA levels between the recovered posttraumatic stress disorder (PTSD) and control groups (β = −0.030, *P* = 0.684). Similarly, after excluding 14 potential outliers, prefrontal CBF levels were significantly lower in the persistent group relative to the control group (β = −0.279, *P* < 0.001), whereas the recovered PTSD group showed no significant difference compared to the control group (β = −0.016, *P* = 0.823). For limbic CBF levels, group comparisons remained consistent after excluding 10 influential observations, with levels significantly higher in both the persistent PTSD (β = 0.167, *P* = 0.017) and recovered PTSD (β = 0.234, *P* = 0.001) groups compared to the control group.

Additionally, we reassessed the relationships between prefrontal GABA and CBF levels within the PTSD group after excluding potentially influential data points, further confirming the stability of the original findings. Specifically, after excluding 11 potential outliers, higher prefrontal GABA levels remained significantly associated with reduced prefrontal CBF levels (β = −0.176, *P* = 0.032). Likewise, the relationship between prefrontal GABA and limbic CBF levels remained consistent with the original findings after excluding 11 outliers (β = −0.129, *P* = 0.114).

Mediation analysis was used to explore whether prefrontal *and/or* limbic CBF levels mediated the relationship between prefrontal GABA levels and PTSD symptom severity. Results indicated that the prefrontal CBF levels significantly mediated this relationship (Fig. 2g, indirect effect = 0.813, bootstrap standard error [SE] = 0.344, *P* = 0.018), suggesting that disrupted neurovascular coupling may underlie PTSD pathology. Conversely, the limbic CBF levels did not serve as a mediator in this relationship (Fig. 2h, indirect effect = 0.017, bootstrap SE = 0.073, *P* = 0.814),

In summary, data from our cross-sectional study comparing individuals with persistent PTSD, those recovered from PTSD, and healthy controls indicate that elevated prefrontal GABA levels may contribute to the exacerbation of PTSD symptoms, potentially through the modulation of prefrontal CBF.

### Prefrontal GABA normalization correlates with improvement in PTSD symptoms (longitudinal clinical study)

We analyzed a separate group of 65 individuals who had experienced trauma (referred to as the trauma-exposed group) and 61 healthy individuals without any trauma exposure (referred to as the healthy control group). This sample was entirely distinct from participants in cross-sectional clinical study, as detailed in Supplementary Table 2. This longitudinal follow-up study aimed to assess acute PTSD to determine whether prefrontal GABA alterations associated with PTSD normalize as symptoms improved over time. We assessed brain outcome measures, including prefrontal GABA levels and normalized resting-state CBF in the prefrontal and limbic areas, at baseline (12 months post-trauma) and follow-up (20 months post-trauma), as shown in Supplementary Fig 1.

Baseline results from this longitudinal clinical study replicated those from the cross-sectional clinical study regarding the link between PTSD-related GABA alterations, prefrontal CBF, and PTSD symptom severity. The trauma-exposed group demonstrated higher prefrontal GABA and lower prefrontal CBF levels than the healthy control group, with elevated limbic CBF levels also observed in the trauma-exposed group (Supplementary Fig. 2a-c and Supplementary Table 5). Similar to results from the cross-sectional clinical study, elevated prefrontal GABA levels in the trauma-exposed group were associated with reduced prefrontal CBF and increased PTSD symptom severity but not with limbic CBF levels (Supplementary Fig. 2d–f and Supplementary Table 6). Re-estimating the linear regression analysis models after excluding potential outliers yielded consistent results. Specifically, after excluding six potentially influential data points, prefrontal GABA levels remained significantly higher in the trauma-exposed group compared to the control group (β = 0.369, *P* < 0.001). Similarly, the exclusion of eight and six potential outliers for prefrontal and limbic CBF levels, respectively, did not alter the original group comparisons (prefrontal CBF levels, β = −0.273, *P* = 0.002; limbic CBF levels, β = 0.250, *P* = 0.007).

Furthermore, within the trauma-exposed group, higher prefrontal GABA levels retained a marginally significant association with reduced CBF levels after excluding four potential outliers (β = −0.240, *P* = 0.071). Likewise, the relationship between prefrontal GABA and limbic CBF levels remained consistent with the original finding after the removing 4 potential outliers (β = −0.099, *P* = 0.475).

**Fig. 3:**
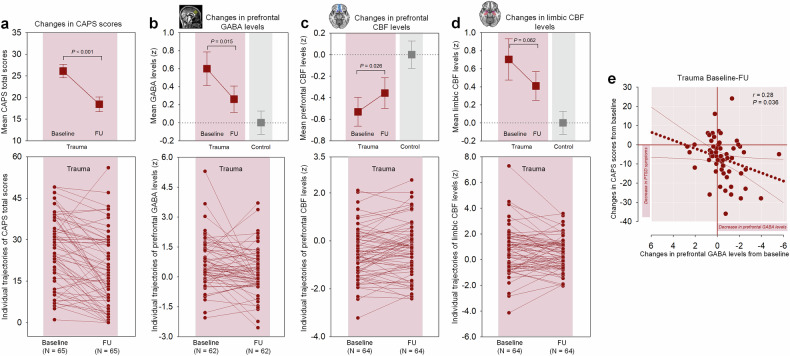
[Longitudinal clinical study] Prefrontal GABA normalization and PTSD symptom improvement in trauma-exposed individuals. **a** PTSD symptom severity, assessed by the CAPS scores, showed significant improvement from baseline to the 8-month follow-up. **b** Prefrontal GABA levels in the trauma-exposed group normalized to the levels observed in the trauma-unexposed group by the time of follow-up assessment. **c** Prefrontal CBF levels increased over time in the trauma-exposed group. **d** Limbic CBF levels in the trauma-exposed group showed a decreasing trend, but the change was not statistically significant. **e** In the trauma-exposed group, decreases in prefrontal GABA levels over time were correlated with an improvement in PTSD symptoms. Error bars in the graphs indicate standard errors of the mean. Abbreviations: CAPS Clinician-Administered Post-traumatic Stress Disorder Scale for DSM-5, FU follow-up, GABA gamma-aminobutyric acid, CBF normalized resting-state cerebral blood flow, PTSD post-traumatic stress disorder

Follow-up assessments were performed to assess the normalization of prefrontal GABA alterations in the trauma-exposed group as symptoms improved. Results indicated a significant reduction in PTSD symptoms during the follow-up (Fig. 3a), with prefrontal GABA levels decreasing to those observed in the healthy control group (Fig. 3b). Furthermore, prefrontal CBF normalized over the follow-up (Fig. 3c). Although limbic CBF levels showed a tendency to decrease, this change was not statistically significant over time (Fig. 3d). Detailed statistical values are provided in Supplementary Table 7. While a significant association was found between the decrease in prefrontal GABA levels and PTSD symptom improvement (Fig. 3e), no significant relationship was observed between changes in prefrontal CBF levels and symptom improvement. These results collectively highlight a significant relationship between the normalization of prefrontal GABA levels and symptom improvement in trauma-exposed individuals.

As part of supplementary analyses, we examined potential differences in prefrontal GABA and CBF levels at follow-up between trauma-exposed individuals who exhibited CAPS score improvement (*N* = 51) and those who did not (*N* = 14). The groups were similar in age (t = 0.18, P = 0.861) and sex distribution (χ2 = 2.58, *P* = 0.108). Baseline CAPS scores were also comparable between the improved (mean ± standard deviation [SD], 27.3 ± 12.4) and non-improved groups (mean ± SD, 22.6 ± 13.4) (t = 1.24, *P* = 0.220), reflecting similar demographic and clinical characteristics at baseline. As expected, follow-up CAPS scores were significantly lower in the improved group (mean ± SD, 15.9 ± 12.5) compared to the non-improved group (mean ± SD, 28.2 ± 14.2) (t = −3.16, *P* = 0.002). However, no significant differences were found in prefrontal GABA levels (β = −0.055, *P* = 0.661), prefrontal CBF levels (β = 0.017, *P* = 0.891), or limbic CBF levels (β = 0.181, *P* = 0.165) between the groups at follow-up. These null results may reflect the limited statistical power due to the small and unbalanced sample sizes (51 vs. 14). Furthermore, the “improved” group was not fully remitted, with 17 individuals still meeting PTSD diagnostic criteria at follow-up, which may have obscured potential differences.

Additionally, we explored whether baseline GABA or CBF levels were associated with changes in CAPS scores, using linear regression analysis adjusted for age and sex. Baseline prefrontal CBF levels (β = 0.004, *P* = 0.975) or prefrontal GABA levels (β = 0.216, *P* = 0.109) showed no significant association with changes in CAPS scores. In contrast, lower baseline limbic CBF levels were significantly linked to greater CAPS score improvement (β = −0.342, *P* = 0.007).

These findings suggest that lower limbic CBF levels at baseline may serve as a potential predictor of greater symptom improvement in trauma-exposed individuals. However, the small sample size and preliminary nature of these results warrant confirmation in future studies with larger, more balanced cohorts.

### Altered prefrontal astrocytic GABA in postmortem brains of PTSD patients (postmortem human brain study)

To investigate the molecular basis of elevated GABA levels observed in PTSD, we analyzed postmortem mPFC tissue from PTSD patients. Previously reported RNA sequencing (RNA-seq) data^25^ revealed a significant upregulation of astrogliosis markers, including glial fibrillary acidic protein (GFAP), monoamine oxidase B (MAOB),^18^ aldehyde Dehydrogenase 1 Family Member L1 (ALDH1L1), acetaldehyde dehydrogenase 2 (ALDH2), and aquaporin 4 (AQP4)^19,26^ (Supplementary Fig. 3a). Given that MAOB and ALDH enzymes are known contributors to astrocytic GABA synthesis^18,27,28^ (Supplementary Fig. 3b). This observation led us to hypothesize the role of aberrant astrocytic GABA in the inhibitory imbalance underlying PTSD.

**Fig. 4:**
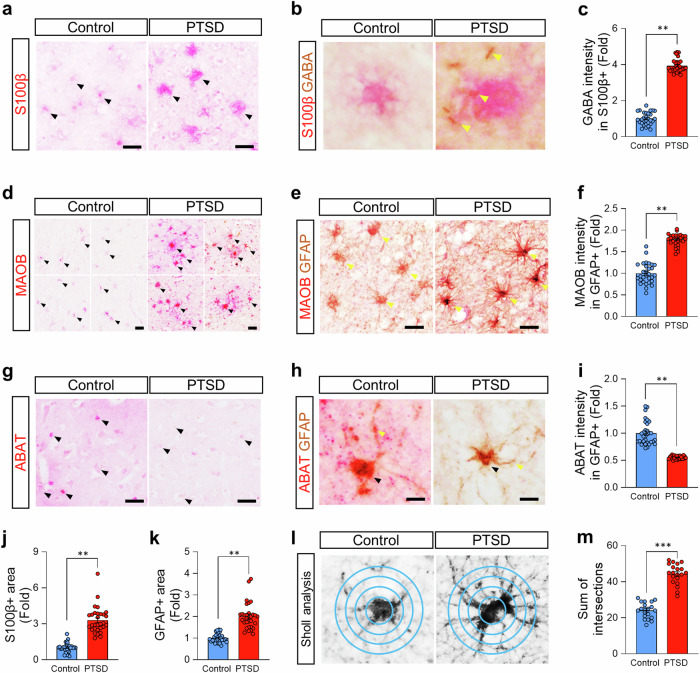
[Postmortem human brain study] Alterations in prefrontal astrocytic GABA in the postmortem brains from PTSD patients. **a**, **b** Representative images of S100β (**a**) and GABA (**b**) in the prefrontal cortices of postmortem brains from a control individual and a patient with PTSD. **c** Quantification of GABA intensity in the S100β-positive area between the control and PTSD groups. **d**, **e** Representative images of MAOB (**d**) and GFAP (**e**) in the prefrontal cortices of postmortem brains from a control individual and a patient with PTSD. **f** Quantification of MAOB intensity in the GFAP-positive area of the control and PTSD groups. **g**, **h** Representative images of ABAT (**g**) and GFAP (**h**) in the prefrontal cortices of postmortem brains from a control individual and a patient with PTSD. **i** Quantification of ABAT intensity in the GFAP-positive areas of the control and PTSD groups. **j** Quantification of the S100β-positive areas of the control and PTSD groups. **k** Quantification of the GFAP-positive areas of the control and PTSD groups. **l** Representative images from the Sholl analysis. **m** Comparison of the sum of the intersections between the control and PTSD groups. Detailed information about the statistical values is provided in Supplementary Table 8. Error bars in the graphs indicate standard errors of the mean. **P < 0.01; ***P < 0.001. PTSD post-traumatic stress disorder, S100β S100 calcium-binding protein B, GABA gamma-aminobutyric acid, MAOB monoamine oxidase B, GFAP glial fibrillary acidic protein, ABAT 4-aminobutyrate aminotransferase

To validate the RNA-seq findings, we performed immunostaining on mPFC tissue from PTSD patients (*N* = 5) and healthy controls (*N* = 5). We analyzed S100β-positive astrocytes from these subjects (n = 30 cells per group) and found a significantly increased GABA intensity in PTSD patients compared to healthy controls (Fig. 4a–c), suggesting an excessive GABA homeostasis in astrocytes. To elucidate the enzymatic mechanism driving this increase, we examined the expression of MAOB^18^ and ABAT,^29^ key enzymes involved in GABA metabolism (Supplementary Fig. 3b). PTSD patients exhibited elevated MAOB intensity (Fig. 4d–f) and reduced ABAT, the enzyme responsible for GABA degradation,^18^ intensity within GFAP-positive astrocytic areas (Fig. 4g–i). This imbalance, characterized by increased GABA synthesis and impaired degradation, suggests a mechanistic explanation for the excessive astrocytic GABA observed in PTSD. These results implicate astrocytic enzyme dysregulation as a critical driver of GABA imbalance, further supporting the role of astrocytes in PTSD pathophysiology.

Further analysis revealed morphological changes in the astrocytes of PTSD patients. Both S100β- and GFAP-positive areas were significantly increased compared to healthy controls (Fig. 4j, k). Sholl analysis, characterized by morphological analysis,^30^ further revealed increased astrocytic branching, indicated by a higher sum of intersections (Fig. 4l, m). These morphological alterations are consistent with reactive astrocytes, aligning with the RNA-seq findings of elevated MAOB and GFAP expression in PTSD and resembling astrocytic alterations observed in other psychiatric disorders.^31^ Together, these results indicate astrocytic GABA dysregulation as a key feature of PTSD, driven by increased MAOB- and ABAT-dependent GABA metabolism, leading us to further investigate the cellular sources of GABA within a PTSD mouse model.

### Astrocytic changes and elevated GABA levels in the IL cortex of a PTSD-like mice model (PTSD-like mouse model study)

**Fig. 5:**
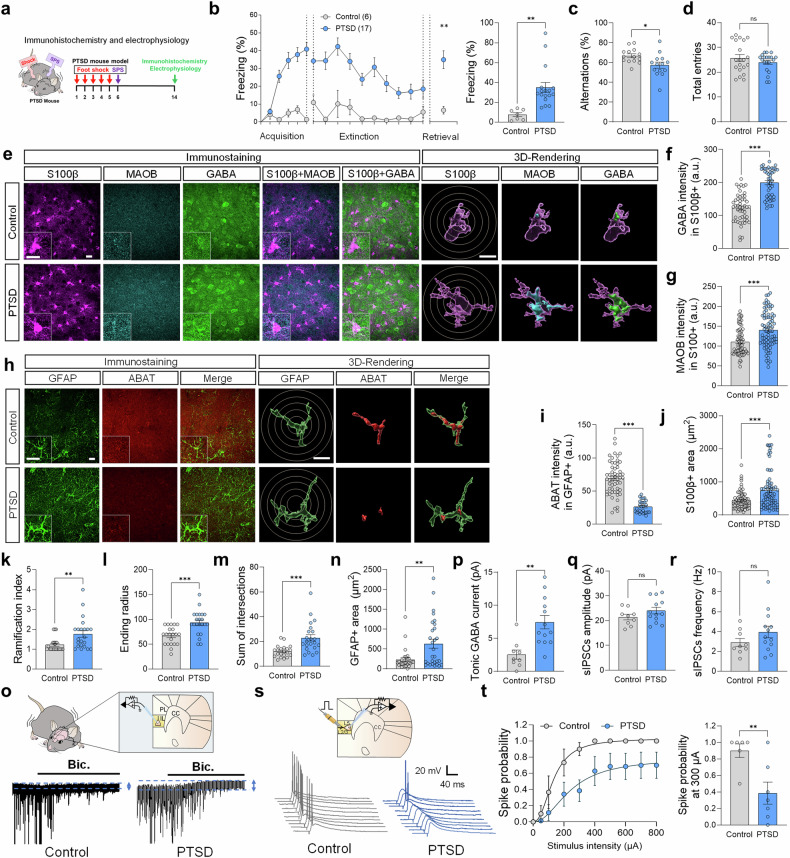
[PTSD mouse model study with KDS2010 treatment] Astrocytic changes and elevated GABA levels in the IL cortex of a PTSD-like mouse model. **a** The timeline for the PTSD-like mouse model and experiments. **b** Contextual fear conditioning, extinction, and extinction retrieval sessions in the control and PTSD-like mouse model. **c**, **d** Y-maze test results showing alternation percentage for spatial working memory (**c**) and total arm entries (**d**). **e** Representative confocal and Imaris images of S100β, MAOB, and GABA in the control and PTSD groups. A circle indicates the Sholl analysis. **f**, **g** Quantification of GABA (**f**) and MAOB (**g**) intensity in the S100β-positive area from confocal images. **h** Representative confocal and Imaris images of GFAP and ABAT in the control and PTSD groups. **i** Quantification of ABAT intensity in the GFAP-positive area. **j** Quantification of S100β-positive area. **k-m** The summary graph shows the ramification index (k), ending radius (**I**), and sum of intersections (**m**) in Sholl analysis. **n** Quantification of GFAP-positive area. **o** A schematic image of a whole-cell patch-clamp recording from an IL cortical neuron and representative traces of tonic GABA currents. **p–r** A summarized graph showing tonic GABA currents (**p**), sIPSC amplitude (**q**), and the frequency (**r**) in IL cortical neurons across the groups. **s** Schematic diagram of spike probability measurements and representative traces of evoked EPSPs from layer II stimulation (*n* = 3 mice per group). **t** Spike probability across stimulation intensities (50–800 μA) (**left**) and comparison of spike probability at 300 μA (**right**). Detailed information about the statistical values is provided in Supplementary Tables 9 and 10. Error bars in the graphs indicate standard errors of the mean. * *P* < 0.05; **P < 0.01; ***P < 0.001. ns not significant. PTSD post-traumatic stress disorder, SPS single prolonged stress, CBF cerebral blood flow, GABA gamma-aminobutyric acid; S100β, S100 calcium-binding protein B, MAOB monoamine oxidase B, ABAT 4-aminobutyrate aminotransferase, GFAP glial fibrillary acidic protein, sIPSC spontaneous inhibitory postsynaptic current, a.u. arbitrary unit, IL infralimbic, CC corpus callosum

Building on these findings, we employed a PTSD-like mouse model to explore further molecular and cellular mechanisms of astrocytic dysfunction in GABA tone. The PTSD-like mouse model was established by subjecting mice to a combination of single prolonged stress (SPS) and inescapable foot shocks, which induce PTSD-related behavioral impairments (Fig. 5a), as previously described.^32^ Behavioral assessments confirmed the development of PTSD-like symptoms, with mice exhibiting impaired fear extinction and retrieval in the contextual fear conditioning (CFC), as indicated by increased freezing behavior during retrieval sessions compared to controls (Fig. 5b). Additional experiments including a footshock-only control group demonstrated that while footshock alone induced normal fear extinction, PTSD-like mice showed significantly impaired extinction retrieval (Supplementary Fig. 4a), further validating the impaired extinction in the PTSD-like model. Similarly, we found that the PTSD mice exhibited deficits in working memory in the Y-maze test (Fig. 5c, d), further validating the PTSD-like mice.

Next, to determine whether the elevated GABA levels observed in PTSD patients are recapitulated in the PTSD-like mouse model, we stained for GABA within S100β-positive astrocytes in the IL cortex. Consistent with the postmortem data, we found a significantly increased GABA in S100β-positive astrocytes in the PTSD-like mouse compared to the control (Fig. 5e, f), indicating an excessive GABA similar to PTSD patients. To further investigate the source of the aberrant GABA, we investigated the expression of enzymes involved in GABA metabolism. We found that MAOB was significantly upregulated in the S100β-positive astrocytes, while ABAT was significantly decreased in GFAP-positive astrocytes (Fig. 5g-i). Furthermore, putrescine levels, a substrate for MAOB-mediated GABA synthesis,^33^ were significantly elevated in PTSD-like mice (Supplementary Fig. 5a, b). These metabolic changes were accompanied by astrogliosis, including increased S100β- and GFAP-positive astrocytic areas and higher ramification indices in the PTSD-like mice compared to the control (Fig. 5j-m and Supplementary Fig. 5c-f). Together, these findings suggest that the aberrant astrocytic GABA in PTSD is possibly driven by enhanced synthesis via MAOB and reduced degradation via ABAT in reactive astrocytes, contributing to the excessive inhibitory tone observed in PTSD.

To determine whether the astrocytic GABA led to increased GABA inhibition in PTSD-like mice, we employed whole-cell patch-clamp recordings from cortical pyramidal neurons in the IL cortex to measure tonic GABA current, as previously established.^34^ We demonstrated that tonic GABA currents, primarily driven by astrocytic GABA,^17^ were significantly higher in PTSD-like mice compared to controls when measured following the administration of a GABAA receptor antagonist, bicuculline (50 µM) (Fig. 5o, p). In contrast, there were no significant differences in the amplitude or frequency of GABAA receptor-mediated spontaneous inhibitory postsynaptic currents (sIPSCs) in the PTSD and control groups (Fig. 5q, r), suggesting that synaptic GABA transmission remained unaffected. We further assessed the consequence of increased tonic GABA inhibition by evaluating the spike probability of layer V neurons in the IL cortex by measuring the spike probability upon electrical stimulation of the layer II/III circuit. Spike probability was significantly reduced in the PTSD-like mouse compared to the control (Fig. 5s, t), suggesting that the elevated astrocytic tonic GABA may inhibit neural activity critical for fear extinction circuits in the IL cortex. This reduced excitability is likely mediated by a shunting mechanism,^16^ whereby increased tonic GABA conductance lowers membrane resistance and dampens depolarization from excitatory inputs without altering EPSP amplitude.

Together, these results demonstrate that aberrant astrocytic GABA in the IL cortex of PTSD-like mice leads to increased tonic inhibition, mirroring the inhibitory imbalance observed in PTSD patients. This model provides a platform for further mechanistic studies aimed at targeting astrocytic MAOB and restoring GABA homeostasis to alleviate PTSD-related symptoms.

### IL astrocytic MAOB is necessary and sufficient for impaired extinction memory in the PTSD-like mice model (Genetic mouse model)

**Fig. 6:**
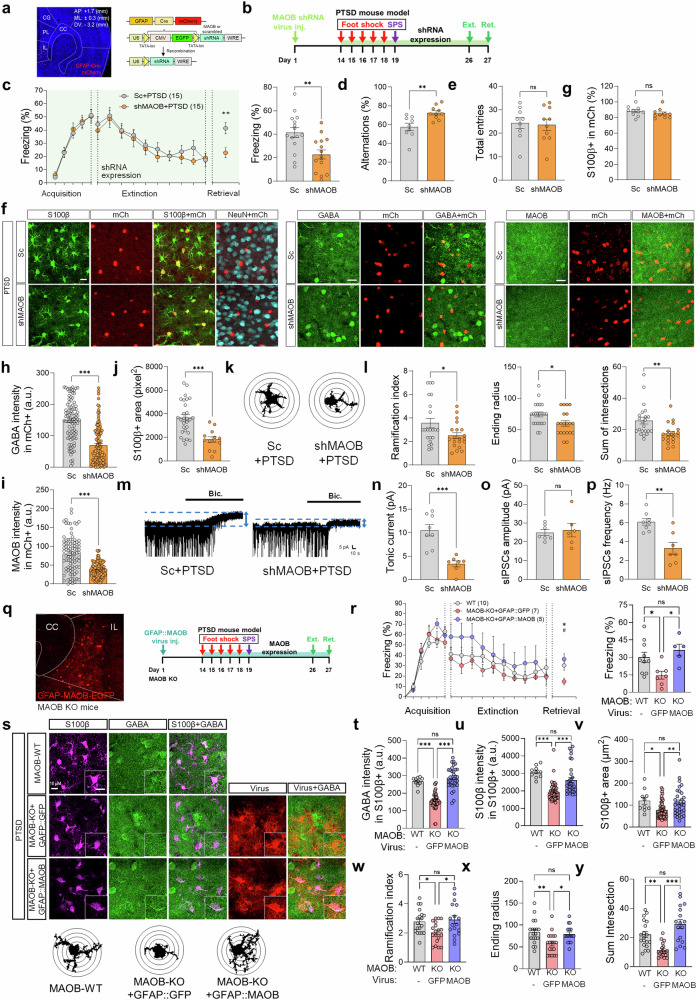
[Genetic mouse model study] Role of IL astrocytic MAOB in modulating extinction memory in a PTSD mouse model. **a** Astrocyte-specific gene-silencing of MAOB in the IL cortex, employing pSico-shRNA with GFAP-Cre virus. **b** Timeline for the development of shMAOB+PTSD and Sc+PTSD mouse models, followed by immunohistochemistry, electrophysiology, and contextual fear conditioning. **c** Contextual fear conditioning, extinction, and extinction retrieval sessions in the Sc+PTSD and shMAOB-PTSD groups. **d**, **e** Y-maze test results showing alternation percentage for spatial working memory (**d**) and total arm entries (**e**). **f** Immunostaining analyses and confocal microscopy images of GABA, MAOB, S100β, and NeuN in the Sc+PTSD and shMAOB+PTSD groups. **g** Quantification of the proportion of S100β-positive cells among mCherry-positive cells in Sc and shMAOB groups showing no significant difference. **h**, **i** Quantification of GABA (**h**) and MAOB (**i**) intensity within the mCherry-positive astrocytic areas across the groups. **j** Quantification of S100β-positive area. **k** Representative Sholl analysis images of astrocytes. **l** The summary graph shows the ramification index (left), ending radius (middle), and sum of intersections (right). **m** Representative traces of tonic GABA currents in the Sc + PTSD and shMAOB + PTSD groups. **n-p** Summarized data on tonic GABA currents (**n**), sIPSC frequency (**o**), and amplitude (**p**) of the IL cortical neuron across the groups. **q** Experimental timeline and schematic representation of MAOB-KO mice expressing GFAP::MAOB or GFAP::GFP (control) for astrocyte-specific expression. **r** Contextual fear conditioning results showing extinction and retrieval performance (**left**) and freezing behavior in the retrieval session (**right**) in MAOB-WT littermates, MAOB-KO + GFAP::GFP and MAOB-KO + GFAP::MAOB groups. **s** Representative images of S100β and GABA staining across groups, including Sholl analysis images for astrocytes. **t**, **u** Quantification of GABA (**t**) and S100β (**u**) intensity in S100β-positive areas. **v** Quantification of S100β-positive areas. **w–y** The summary graph shows the ramification index (**w**), ending radius (**x**), and sum of intersections (**y**) in Sholl analysis. Detailed information about the statistical values is provided in Supplementary Tables 11,12, and 13. Error bars in the graphs indicate standard errors of the mean. *P < 0.05; **P < 0.01; ***P < 0.001; ns, not significant. AAV adeno-associated virus, Lenti lentivirus, IL infralimbic PL prelimbic, CG anterior cingulate CC corpus callosum, AP anterior-posterior, ML medial-lateral, DV dorsal-ventral, MAOB monoamine oxidase B, KD knockdown, KO knockout, WT wild type, PTSD post-traumatic stress disorder, SPS single prolonged stress, shRNA single hairpin RNA, GFAP glial fibrillary acidic protein, Sc scramble shRNA, shMAOB MAOB shRNA, GABA gamma-aminobutyric acid, mCh mCherry; a.u., arbitrary unit, S100β S100 calcium-binding protein B, sIPSC spontaneous inhibitory postsynaptic current

Next, to determine whether the astrocytic MAOB is necessary for PTSD symptoms, we employed astrocyte-specific MAOB knockdown in the IL cortex of the PTSD-like mouse model. Two weeks before PTSD modeling, we co-injected a GFAP-Cre-mCherry with pSico-scrambled (Sc) short hairpin RNA (shRNA) or MAOB shRNA (shMAOB) into the IL cortex, generating the Sc+PTSD and shMAOB+PTSD groups, respectively (Fig. 6a, b). We found that the inhibition of astrocytic MAOB significantly improved the fear extinction retrieval, as evidenced by reduced freezing behavior in the shMAOB+PTSD group compared to the Sc+PTSD group (Fig. 6c). In contrast, inhibiting astrocytic MAOB in the PL cortex did not affect the fear extinction retrieval (Supplementary Fig. 6), underscoring the specific role of IL astrocytes in modulating fear extinction memory. Furthermore, we found that the shMAOB+PTSD group showed improved working memory in the Y-maze test (Fig. 6d, e) without affecting locomotor activity in the open field (Supplementary Fig. 7a). These findings suggest that astrocytic MAOB in the IL cortex is responsible for fear extinction retrieval and working memory.

We then investigated the cellular consequences of astrocytic MAOB inhibition in the PTSD-like mouse model. To validate the specificity and efficiency of our viral strategy for astrocyte-targeted MAOB knockdown, we performed colocalized analysis for mCherry, S100β, and NeuN (Fig. 6f). Quantitative analysis demonstrated that the vast majority of mCherry-positive cells colocalized with S100β, while less than 1% overlapped with NeuN-positive neurons in both the Sc+PTSD and shMAOB+PTSD group (Supplementary Fig. 8). This indicates that mCherry expression was highly selective for astrocytes. Furthermore, we found that approximately 88% of S100β-positive astrocytes in the Sc+PTSD and 85% in the shMAOB+PTSD groups were also mCherry positive (Fig. 6f, g), indicating high infection efficiency across astrocytes populations. Together, these results confirm the astrocyte-selective targeting of our viral construct.

The shMAOB+PTSD group exhibited reduced GABA and MAOB intensity in mCherry-positive astrocytic areas compared to the Sc+PTSD group (Fig. 6f–i), confirming the effective MAOB knockdown. Notably, the shMAOB+PTSD group showed a significant decrease in S100β-positive areas and Sholl analysis indices (Fig. 6j–l). Furthermore, tonic GABA current was significantly reduced in the shMAOB+PTSD group, while sIPSC amplitude showed no difference, and frequency exhibited a decrease in the shMAOB+PTSD group (Fig. 6m–p). These findings collectively suggest that PTSD-related GABA dysregulation may stem from astrocytes and that MAOB is necessary for PTSD symptoms.

Beyond the necessity, we tested whether MAOB-dependent astrocytic GABA in the IL of PTSD-like mice is sufficient for impaired fear extinction retrieval within a PTSD context. Two weeks before PTSD modeling, we selectively overexpressed MAOB in the IL cortex astrocyte of MAOB null knockout (MAOB-KO)^35^ mice using the AAV-GFAP::MAOB-EGFP virus in IL of PTSD-like mouse, creating the MAOB-overexpression (MAOB-OE) + PTSD group (Fig. 6q). For the control, we used the AAV-GFAP::GFP virus, resulting in the MAOB-KO + PTSD group. Consistent with the gene-silencing results, we found that MAOB-KO + PTSD showed a significantly lower fear response in extinction retrieval compared to MAOB wild-type littermate mice (MAOB-WT) + PTSD (Fig. 6r). Conversely, the MAOB-OE + PTSD group significantly increased freezing behavior during fear memory retrieval compared to the MAOB-KO + PTSD group (Fig. 6r), suggesting the sufficiency of astrocytic MAOB in this process.

We next demonstrated that aberrant astrocytic GABA in the MAOB-WT group was significantly decreased in the MAOB-KO + PTSD group (Fig. 6s, t). However, astrocytic GABA levels in the MAOB-OE + PTSD group were similar to those in the MAOB-WT + PTSD group (Fig. 6s, t). Also, MAOB-KO + PTSD groups exhibited decreased S100β-positive areas, S100β intensity, and astrogliosis compared to both MAOB-WT + PTSD and OE + PTSD groups (Fig. 6u-y). Altogether, these findings demonstrate the necessity and sufficiency of IL astrocytic MAOB in disrupting fear extinction retrieval, leading us to investigate whether pharmacological inhibition of MAOB could provide a therapeutic strategy for PTSD.

### Pharmacological inhibition of MAOB in PTSD-like mice model (MAOB-inhibitor drug therapy)

To evaluate whether pharmacological inhibition of MAOB could reverse PTSD-related symptoms, we utilized KDS2010^36^, a potent, selective, and reversible MAOB inhibitor. KDS2010 was designed to overcome the limitation of existing MAO inhibitors, which are often non-selective (targeting both MAOA and MAOB) and irreversible, leading to compensatory side effects with long-term use.

**Fig. 7:**
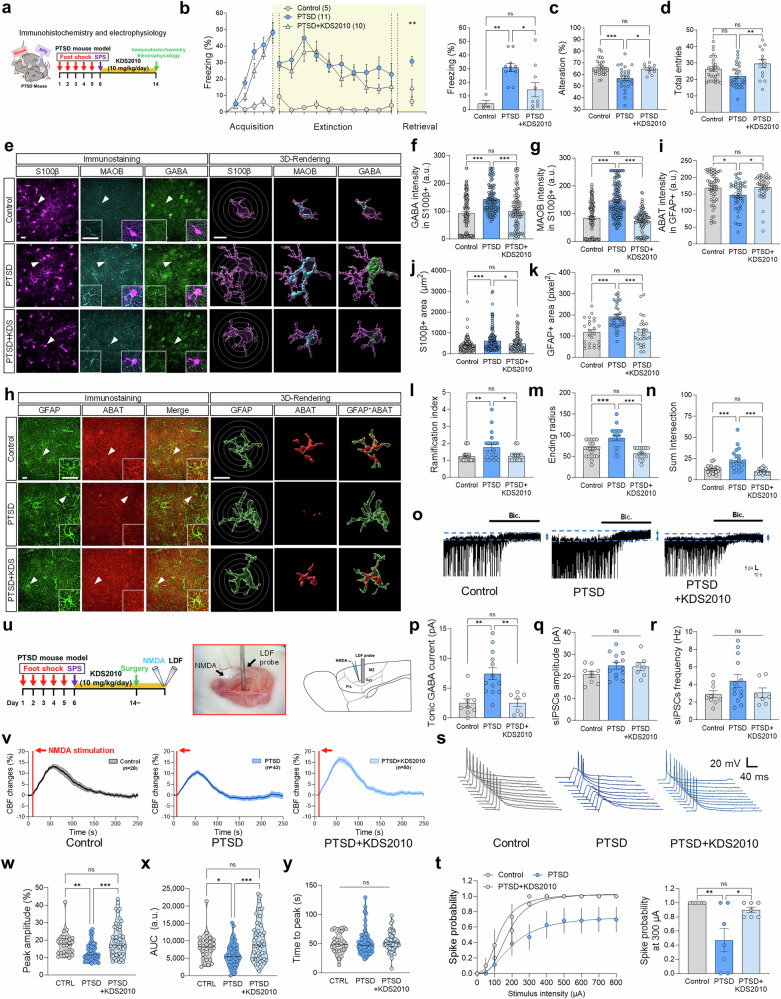
[PTSD mouse model study with KDS2010 treatment] Effects of MAOB-dependent tonic GABA alterations on CBF and fear extinction in the PTSD mouse model and their reversal through pharmacological MAOB inhibition. **a** The timeline for the PTSD-like mouse model KDS2010 (10 mg/kg/day, ad libitum drinking) treatments, and behavior experiments. **b** Behavioral data from the contextual fear conditioning, extinction, and extinction retrieval in the control, PTSD-like mouse, and PTSD-like mouse+KDS2010 groups. **c, d** Y-maze test results showing alternation percentage for spatial working memory (**c**) and total arm entries (**d**). **e** Representative confocal and Imaris images of S100β, MAOB, and GABA in the control, PTSD, and PTSD + KDS2010 groups (arrows indicate the location of inset images). Circle indicates Sholl analysis of astrocytes. **f, g** Quantification of GABA (**f**) and MAOB (**g**) intensity in the S100β-positive areas. **h** Representative confocal images of GFAP and ABAT in the control, PTSD, and PTSD + KDS2010 groups. **i** Quantification of ABAT intensity in the GFAP-positive areas. **j, k** Quantification of S100β- (**j**) and GFAP- (**k**) positive area. **l-n** Quantification of ramification index (**l**), ending radius (**m**), and sum of intersection (**n**). **o** Representative traces of tonic GABA currents across the groups. **p-r** A summarized graph showing tonic GABA currents (**p**), sIPSC amplitude (**q**), and the frequency (**r**) in IL cortical neurons. **s** Schematic diagram of spike probability measurements and representative traces of evoked EPSPs (*n* = 3 mice per group). **t** Spike probability across stimulation intensities (50-800 μA) (**left**) and comparison of spike probability at 300 μA (**right**). **u** The timeline for the PTSD-like mouse model, LDF recording, and NMDA stimulation for CBF measurement (**left**). Representative image of LDF probe installation and NMDA application in the frontal cortex (**middle, right**). **v** A representative trace of NMDA stimulation-evoked CBF changes for each group. **w–y** Violin graphs depicting the peak amplitude (**w**), AUC (**x**), and time to peak (**y**) NMDA stimulation–evoked CBF changes in the control, PTSD, and PTSD + KDS2010 groups. Detailed information about the statistical values is provided in Supplementary Table 13. Error bars in the graphs indicate standard errors of the mean. *P < 0.05; **P < 0.01; ***P < 0.001; ns, not significant. PTSD post-traumatic stress disorder, SPS single prolonged stress, CBF cerebral blood flow, NMDA, N-methyl-D-aspartate, LDF Laser Doppler Flowmetry, GABA gamma-aminobutyric acid, S100β S100 calcium-binding protein B, MAOB monoamine oxidase B, ABAT 4-aminobutyrate aminotransferase, GFAP glial fibrillary acidic protein, sIPSC spontaneous inhibitory postsynaptic current, a.u. arbitrary unit, AUC area under the curve, IL infralimbic, CC corpus callosum

PTSD-like mice were treated with KDS2010 administration (10 mg/kg/day, *ad libitum* drinking water) starting on day 6 post-stress exposure (Fig. 7a). Behaviorally, KDS2010 treatments significantly improved fear extinction retrieval in the PTSD-like mice (Fig. 7b). Similarly, Spatial working memory deficit was also restored to control levels in KDS2010-treated mice (Fig. 7c, d) as measured by the Y-maze test, without affecting total locomotor activity in the open field test (Supplementary Fig. 7b). These results suggest the therapeutic potential of targeting MAOB-dependent astrocytic GABA for PTSD treatment. To investigate the underlying mechanism of these behavioral improvements, we analyzed astrocytic GABA and electrophysiological changes. We demonstrated that the excessive astrocytic GABA levels in the IL cortex of PTSD-like mice were fully restored to control levels (Fig. 7e, f), while neuronal GABA levels, although slightly elevated, remained unaffected by KDS2010 (Supplementary Fig. 9a, b). Moreover, KDS2010 reversed the increased astrocytic MAOB expression and decreased ABAT levels in the PTSD-like model (Fig. 7e–i), indicating a restoration of GABA homeostasis in the astrocytes. Consistent with these findings, markers of astrogliosis, including S100β- and GFAP-positive areas, which were significantly increased in PTSD-like mice, were reduced to control levels by KDS2010 treatment (Fig. 7j-n). Interestingly, although KDS2010 did not reduce elevated astrocytic putrescine levels in PTSD-like mice, suggesting that it does not suppress upstream sources of putrescine synthesis, the restoration of MAOB-dependent GABA homeostasis by KDS2010 may still be sufficient to achieve behavioral recovery (Supplementary Fig. 9e, f). Electrophysiological recordings further showed that KDS2010 normalized excessive tonic GABA currents and synaptic inhibition in PTSD-like mice, while synaptic GABA inhibition remained unchanged (Fig. 7o–t), underscoring its selective impact on tonic inhibition. Notably, no differences were observed in NeuN-positive areas or cell numbers across all groups (Supplementary Fig. 9c, d).

Given that enhanced dopaminergic transmission has been implicated in alleviating fear extinction,^37^ we sought to determine whether KDS2010 also affects dopamine dynamics in PTSD-like mice. To this end, we monitored striatal dopamine using AAV-hSyn-GRABDA2m sensors. Evoked dopamine was moderately reduced in PTSD-like mice compared to the controls, and the KDS2010 treatment significantly restored dopamine response compared to PTSD-like mice (Supplementary Fig. 10). These findings suggest that the aberrant tonic GABA inhibition observed in PTSD-like mice may suppress dopaminergic presynapse and that KDS2010 promotes dopamine recovery by relieving the inhibition.

We then examined whether the tonic GABA is likely linked with CBF level in the PTSD-like mice model. To investigate the CBF levels of the PFC in control, PTSD, and PTSD + KDS2010 mice, we employed the Laser Doppler flowmetry (LDF) recording (Fig. 7t) as previously described.^38,39^ For LDF recording, we used NMDA (N-methyl-D-aspartate) stimulation to evoke activity-dependent CBF changes in the PFC of PTSD mice (Fig. 7u). Aligned with the human CSF experiments, we found that PTSD-like mice showed significant decreases in NMDA-evoked CBF and area under the curve (AUC) compared to the control, while time to peak was not significant (Fig. 7v–y). On the other hand, an administration of KDS2010 in PTSD-like mice revealed the NMDA-evoked CBF to the control levels in peak amplitude and AUC (Fig. 7v–y), linking astrocytic GABA dysregulation with impaired neurovascular coupling. These results suggest that CBF levels might be associated with MAOB-dependent GABA in the PTSD-like mice model. Altogether, these results indicate that pharmacological inhibition of MAOB with KDS2010 effectively mitigates PTSD-associated deficits by reducing aberrant astrocytic GABA, restoring behavioral functions, and supporting the therapeutic potential of MAOB inhibitors in PTSD treatment.

## Discussion

In this study, we demonstrated the critical role of the prefrontal GABA in the development of and recovery from PTSD in human subjects. Large-scale human investigations, including cross-sectional and longitudinal analyses,^7^ revealed that elevated GABA levels and reduced CBF in the PFC were associated with more severe symptoms and poorer recovery trajectories (Study 1).^8^ In contrast, trauma-exposed individuals who recovered showed normalized GABA and CBF over time, correlating with symptom improvement (Study 2).^7,8^ Postmortem analyses further identified astrocytes as the source of this dysregulation, with increased MAOB-mediated GABA synthesis and reduced ABAT-mediated degradation as key underlying mechanisms (Study 3).^40,41^ Complementary mouse studies extended these findings, demonstrating that reactive astrocytes, characterized by hypertrophy and elevated MAOB expression, produced excessive tonic GABA inhibition, impairing fear extinction retrieval (Study 4). Importantly, genetic and pharmacological inhibition of MAOB using KDS2010, a selective and reversible MAOB inhibitor, effectively restored GABA homeostasis, normalized tonic inhibition, and enhanced fear extinction retrieval without altering synaptic GABA activity (Studies 5, 6). Although variability in freezing responses was observed among PTSD-like mice, KDS2010 treatment consistently improved extinction retrieval across the groups (Supplementary Fig. 4b). By integrating human clinical studies, postmortem analyses, and preclinical mouse models, this study establishes a mechanism in which astrocytic GABA dysregulation disrupts prefrontal circuits, impairs fear extinction, and modulates CBF. These findings highlight MAOB inhibition as a promising therapeutic target to restore prefrontal inhibitory balance and alleviate PTSD symptoms (Supplementary Fig. 11).

In this study, we observed a distinct pattern of CBF alterations in the prefrontal and limbic regions, suggesting a region-specific mechanism of dysfunction. Reduced prefrontal CBF was closely associated with elevated GABA levels and PTSD symptom severity, consistent with prior studies linking prefrontal hypoperfusion to impaired inhibitory control and deficits in fear extinction.^42^ In contrast, limbic CBF remained elevated, aligning with reports implicating hyperactivity in the amygdala and related limbic regions in heightened emotional reactivity and stress responses. These findings indicate that prefrontal tonic GABA-mediated inhibition may impair extinction by suppressing neuronal excitability and metabolic demand, whereas persistent limbic hyperactivity may operate through distinct mechanisms. Future studies examining connectivity between the PFC, amygdala, and hippocampus will be critical for understanding network-level interactions in PTSD. These insights could clarify whether restoring CBF alone is sufficient for symptom recovery or if targeting astrocytic GABA remains essential for addressing inhibitory dysfunction.

While our findings consistently point to reactive astrocytic GABA dysregulation as a key pathological feature of PTSD, the nature of the astrocytic response to stress appears to diverge across psychiatric conditions.^43,44^ In chronic stress-induced depression models, astrocytes often undergo morphological atrophy and functional asthenia, including impaired glutamate clearance, reduced neurotrophic support, and retraction of fine perisynaptic processes.^44,45^ These features are frequently accompanied by the downregulation of ezrin,^46^ a cytoskeletal protein critical for maintaining astrocytic leaflets and synaptic coverage. In contrast, our PTSD-like model, which combined footshock and single prolonged stress, induced astrocyte hypertrophy, elevated MAOB expression, and increased tonic GABA release. These findings are consistent with the reactive astrogliosis observed in postmortem PTSD tissue and suggest that functional asthenia may also present in the form of excessive astrocytic inhibition, leading to reduced circuit excitability and impaired fear extinction. The contrasting astrocytic phenotypes observed in depression and PTSD models likely reflect differences in stress modality, duration, and affected brain regions. Further studies incorporating markers such as ezrin, along with assessments of both glutamate and GABA metabolism, will be interesting to determine whether these represent distinct pathological states or alternative expressions of astrocytic dysfunction under stress.

Previous studies have suggested that fear extinction may also be improved by increased dopamine,^37^ raising the possibility that dopaminergic mechanisms could contribute to PTSD pathology. Although MAOB has been described as a dopamine-degradation enzymes,^47^ we have recently shown that MAOA primarily degrades dopamine, whereas MAOB selectively regulates tonic GABA inhibition in the striatal neurons.^48^ Similar to the previous findings, our dopamine imaging results revealed that PTSD-like mice exhibited reduced striatal dopamine release, which was restored to control levels following KDS2010 treatment. These observations suggest that excessive astrocytic GABA may suppress presynaptic dopamine signaling in PTSD conditions. Future studies will be necessary to delineate the possibility.

Clinical evidence supports the therapeutic effects of selective MAO inhibitors, such as selegiline, which exert a therapeutic effect in a variety of neuropsychiatric diseases.^49,50^ These findings validate MAOB as a promising therapeutic target, consistent with our findings that MAOB-mediated astrocytic GABA synthesis contributes to PTSD pathophysiology. However, long-term use of irreversible MAO inhibitors has been associated with compensatory mechanisms, such as the upregulation of diamine oxidase (DAO),^36^ which can limit long-term efficacy. In contrast, KDS2010,^36^ a reversible and selective MAOB inhibitor, overcomes these limitations by providing sustained inhibition without triggering compensatory enzyme activation. Our study demonstrated that KDS2010 effectively targets MAOB-mediated astrocytic GABA synthesis, reducing tonic inhibition and enhancing fear extinction retrieval, positioning it as a promising therapeutic intervention for PTSD.

Preclinical studies of KDS2010 have demonstrated its pharmacological efficacy and favorable safety profile, supporting its translation to human studies. Recent Phase 1 clinical trial results confirmed its tolerability and pharmacologically active exposure levels in healthy participants (AUCinf 13850.7 ng·h/mL and doses up to 480 mg).^29^ Building on these, a Phase 2 is ready to evaluate the potential efficacy of KDS2010 in PTSD, offering a promising therapeutic approach targeting astrocytic GABA dysregulation. This translational pipeline highlights KDS2010 as a first-in-class therapy, bridging preclinical insights to clinical applications and establishing astrocyte-targeted interventions as a novel paradigm in PTSD treatment.

Despite these promising findings, future investigations should examine whether polyamine metabolism and pathways like the urea cycle influence GABA homeostasis^16^ and contribute to tonic inhibition in PTSD. Astrocytes contain a functional urea cycle, where the enzyme ornithine decarboxylase 1 (ODC1) converts ornithine into putrescine, providing a precursor for MAOB-dependent GABA.^30,33^ Inhibition of ODC1 could block putrescine production, thereby reducing GABA synthesis and restoring inhibitory balance, suggesting another potent therapeutic strategy for PTSD. These findings underscore the potential for developing additional therapeutic targets beyond MAOB inhibition. Expanding analyses to other PTSD-relevant brain regions, such as the hippocampus and amygdala, will also provide insights into network-level dynamics and cross-regional interactions.

Furthermore, although significant group-level differences in GABA and CBF were observed in our human studies even after controlling for sex, the higher prevalence of PTSD in women and potential sex-based neurobiological differences may influence treatment efficacy. Previous studies have shown that enhancing GABAergic neurotransmission reduced conditioned freezing in female PTSD-like mice.^51,52^ Although our preclinical studies mostly employed male mice, this choice was made to minimize variability, particularly because MAOB is located on the X chromosome, making males a more straightforward model for assessing MAOB effects without the potential confound of X-inactivation. Importantly, our MAOB knockout experiments included both male and female mice, and no significant sex differences were observed in these analyses. Future research should incorporate female animal models to investigate potential sex-specific responses to MAOB inhibition, which will be essential for fully understanding the translational implications of astrocytic GABA dysregulation in PTSD across sexes. However, we acknowledge that all postmortem brain samples used in this study were obtained from male subjects due to limitations in available matched tissue. This constitutes a potential limitation in terms of generalizability, as sex-dependent pathological features in PTSD remain incompletely characterized. Future studies incorporating female postmortem samples will be necessary to elucidate potential sex-specific neurobiological mechanisms.

It is important to note that our human sample in the cross-sectional and longitudinal studies lacked a trauma-exposed non-PTSD group. The cross-sectional clinical study included three groups: a healthy control group with no trauma exposure, a persistent PTSD group, and a recovered PTSD group. The longitudinal clinical study included two groups: a healthy control group with no trauma exposure and a trauma-exposed group, the latter consisting of individuals with a current PTSD diagnosis (*N* = 41) and those with subclinical PTSD symptoms but no formal diagnosis (*N* = 24, with a mean CAPS total score of 12.7). Although the recovered PTSD group in the cross-sectional clinical study may share some characteristics with a trauma-exposed non-PTSD group, the inclusion of this critical control group—alongside a no-trauma-exposed control group and a trauma-exposed PTSD group—is essential for disentangling the effects of trauma exposure itself from the pathophysiological processes specific to PTSD.^53^ Moreover, comparing individuals who experience similar traumatic events but do not develop PTSD with those who do can provide valuable insights into protective and resilience mechanisms that mitigate the onset of posttraumatic symptoms.^54^ Thus, future studies employing a three-group design are necessary to isolate trauma-related changes independent of disorder-specific effects and to identify moderators and mediators of resilience.

In conclusion, our study identifies astrocytic GABA dysregulation as a central mechanism in PTSD pathophysiology and demonstrates that targeting MAOB inhibition restores inhibitory balance and alleviates symptoms. By integrating human clinical studies, postmortem analyses, and preclinical models, these findings position KDS2010 as a promising therapy, offering a novel paradigm for PTSD treatment and improving clinical outcomes in PTSD.

## Materials and methods

### Two large-scale human studies

#### Study samples and designs

##### Cross-sectional clinical study

The study population comprised three distinct groups: 78 individuals with ongoing PTSD (*persistent PTSD group*), 84 individuals who had previously been diagnosed with PTSD but had recovered (*recovered PTSD group*), and 86 healthy individuals who had no exposure to an index trauma (*healthy control group*). The index trauma was defined as either 1) a direct experience of a life-threatening event or 2) learning of the violent death or life-threatening accident of a closely related person. The persistent and recovered PTSD groups were collectively categorized as the *PTSD group* for certain analyses. Exclusion criteria included significant medical or neurological conditions, major psychiatric disorders, a history of traumatic brain injury with loss of consciousness, and contraindications to brain MRI.

Cross-sectional evaluations involving clinical assessment and multimodal neuroimaging were conducted across all groups (Supplementary Fig. 1a). For those in the persistent and recovered PTSD groups, PTSD diagnosis and symptom severity were assessed using the Clinician Administered PTSD Scale for Diagnostic and Statistical Manual of Mental Disorders, fifth edition (CAPS).^55^

##### Longitudinal clinical study

Participants in this longitudinal follow-up study were entirely distinct from those in the above cross-sectional study. This study included 65 individuals recently exposed to an index trauma (*trauma-exposed group*) and 61 healthy individuals without trauma exposure (*healthy control group*). The exclusion criteria remained consistent with cross-sectional clinical study. Both groups underwent clinical and multimodal neuroimaging assessments at baseline (12 months since trauma exposure). Additionally, the trauma-exposed group received a follow-up assessment approximately 8 months after the baseline assessment (20 months since trauma exposure, Supplementary Fig. 1).

Participant demographics and characteristics for all human studies are detailed in Supplementary Table 1 and 2, respectively. The study protocols were approved by the Institutional Review Board of Ewha Womans University (IRB No: 81-11). All study participants provided written informed consent prior to enrollment.

### Brain image data acquisition in humans

All multimodal MRI and MRS data were collected using a 3.0 Tesla Philips MR scanner system (Philips Healthcare, Best, The Netherlands) equipped with a 32-channel head coil. High-resolution three-dimensional T1-weighted images were obtained through a three-dimensional turbo field echo sequence. The acquisition parameters were as follows: echo time (TE) of 3.4 ms, repetition time (TR) of 7.4 ms, flip angle of 8°, field of view (FOV) of 220 × 220 mm, slice thickness of 1 mm, and 180 contiguous sagittal slices. The volumetric T1-weighted images were used for spectroscopic voxel placement and tissue segmentation.

For the quantification of prefrontal GABA levels, a Mescher-Garwood Point Resolved Spectroscopy (MEGA-PRESS) sequence was employed, utilizing a simultaneous spectral editing technique. The sequence parameters included a TE of 80 ms, TR of 2,000 ms, water suppression using a multiple optimized insensitive suppression train, pulse duration of 20 ms, an estimated water peak of 4.68 ppm, editing pulses interleaved at 1.9 ppm (ON) and 1.5 ppm (OFF), averaging two signals, a phase cycle of 16, frequency stabilization, and a total of 160 dynamic scans. Automated shimming adjusted the second-order shim currents to minimize field inhomogeneities.

CBF measurements in the prefrontal and limbic ROIs in the cross-sectional clinical study were obtained using ASL perfusion MRI. A pseudocontinuous ASL (pCASL) technique was applied using a three-dimensional single-shot gradient and spin echo (3D-GRASE) sequence with the parameters: TE of 12 ms, TR of 3772 ms, FOV of 220 × 220 mm, voxel size of 2.75 × 2.75 mm, slice thickness of 6 mm, 22 slices, a labeling duration of 1800 ms, and a post labeling delay of 1600 ms. For the longitudinal clinical study, ASL perfusion MR images were obtained using a pCASL single-shot echo-planar imaging sequence with the following parameters: TE of 11 ms, TR of 4000 ms, FOV of 220 × 220 mm, voxel size of 2.75 × 2.75 mm, slice thickness of 6 mm, 18 slices, a labeling duration of 1650 ms, a post labeling delay of 1600 ms, and 40 control-label pairs. To estimate the equilibrium magnetization of arterial blood, an echo-planar imaging proton density (M0) image was acquired without labeling or background suppression using the same acquisition parameters as pCASL scan, except for TR was adjusted.

### Measurement of prefrontal GABA levels in humans

MEGA-PRESS spectra were acquired from a 30 × 30 x 30 mm^3^ voxel of interest (VOI) positioned over the frontal cortex. The VOI placement, detailed in Supplementary Fig. 1b, was carefully chosen to align with the midline of the dorsal anterior cingulate cortex. The ventral-anterior edge of the VOI was aligned parallel to the genu of the corpus callosum, ensuring avoidance of cerebrospinal fluid (CSF) signal inclusion, consistent with prior research methodologies.^56,57^ To ensure consistent voxel placement across the baseline and follow-up assessments in the longitudinal clinical study, baseline voxel placement screenshots and gyral patterns were utilized as reference points for subsequent follow-up scans. GABA level measurements were normalized to water within the same voxel, employing standard parameters for spectral registration, including frequency and phase corrections.^58^ The ‘ON’ and ‘OFF’ editing pulses of each spectrum were then subtracted to enable water suppression and to isolate edited GABA spectra, expressed in institutional units (IUs).

The processing and analysis of ^1^H-MRS data were conducted using the GABA Analysis Toolkit (Gannet, version 3.0)^59^ implemented in MATLAB R2017b (MathWorks Inc., Natick, USA). Initially, ^1^H-MRS data were converted from the original time-domain format provided by the scanner to a frequency-domain GABA-edited spectrum using GannetLoad function. The GABA signal was then isolated by fitting the spectral differences to the edited GABA peak at 3 ppm via nonlinear least-squares fitting using GannetFit, with creatine (Cr) serving as an internal standard for GABA quantification. This estimated GABA concentration was co-registered to the respective T1-weighted image using GannetCoRegister, facilitating the creation of a binary mask of the VOI matching the T1-weighted image geometry. GannetSegment, integrated with Statistical Parametric Mapping 8 (Wellcome Trust Centre for Neuroimaging), was then applied for tissue segmentation. This process segmented the VOI of each T1-weighted image into gray matter, white matter, and CSF fractions, allowing for tissue-corrected GABA quantification.

Visual inspection was conducted for each spectrum, excluding poorly fitted spectra from analysis. Spectra with a normalized fitting residual for GABA below 10% were deemed acceptable. In the cross-sectional clinical study, three spectra were excluded based on these criteria (one from the healthy control group and two from the persistent PTSD group). In the longitudinal clinical study, eight spectra were excluded (two from the healthy control group, three from the trauma-exposed group’s baseline assessment, and three from the follow-up assessment). Tissue-corrected GABA concentrations were utilized as the metric for GABA levels in all analyses.

### Measurement of CBF within the prefrontal and limbic ROIs in humans

Preprocessing and analyses of the ASL perfusion MR images were conducted using the FMRIB Software Library tools (FSL, http://www.fmrib.ox.ac.uk/fsl). The preprocessing involved several steps: skull stripping, motion correction, pairwise subtraction of control and label ASL images, and averaging to derive mean perfusion-weighted images. CBF quantification at the voxel level was achieved by estimating the equilibrium magnetization of arterial blood flow using a saturation inversion recovery of the mean CSF magnetization (M0) images.^60^ We utilized a variational Bayesian approach for calibrating perfusion parameters and assessing the goodness of fit, employing the Bayesian Inference for Arterial Spin Labeling (BASIL) tool (http://fsl.fmrib.ox.ac.uk/fsl/fslwiki/BASIL).^61^ This process generated calibrated images, which were then quantified into absolute CBF maps using a single compartment model, as detailed in prior research.^61^ These maps were normalized against the whole brain’s mean of CBF to account for subject-level variation. The normalized resting-state CBF values were subsequently utilized in the analyses. Visual inspection was conducted to identify and exclude images of poor quality. In the cross-sectional clinical study, six images were excluded due to quality concerns, with three from the healthy control group, one from the recovered PTSD group, and two from the persistent PTSD group. In the longitudinal clinical study, two images from a single trauma-exposed subject were excluded (one from the baseline assessment and one from the follow-up).

In this study, we specifically targeted the prefrontal and limbic regions due to their established roles in fear circuitry.^1,7,8^ and their potential responsiveness to modulation by prefrontal GABA. ROI masks for these areas were predefined using the Automated Anatomical Labeling (AAL) atlas. Specifically, the prefrontal regions encompassed the orbitofrontal and subcallosal cortices, while the limbic region included the amygdala and hippocampus (Supplementary Fig. 1b). CBF values within these ROIs were extracted for each participant in native ASL space through a two-step registration process. Initially, individual calibrated and normalized CBF maps in ASL space were aligned to the corresponding T1-weighted images via affine transformation. Subsequently, these T1 images were non-linearly registered to the Montreal Neurological Institute (MNI) space. We then warped the a priori defined prefrontal and limbic ROIs, initially established in MNI space, back into subject-specific ASL space using the inverse transformation matrices obtained from this two-step registration process. Additionally, T1-weighted images were segmented into gray matter partial volume images using the FSL FMRIB’s Automated Segmentation Tool (FAST).^62^ These individual gray matter images were registered to the corresponding calibrated CBF images using inverse transformation. Using a gray matter probability threshold of 0.4, subject-specific gray matter masks were generated to ensure the extraction of CBF values predominantly from gray matter within the designated ROIs.

### Statistical analysis

Demographic and clinical characteristics across groups were assessed using independent-samples t-tests, analysis of variance (ANOVA), and chi-square tests, or Fisher’s exact test, as appropriate. To facilitate meaningful comparison with the healthy comparison group and quantify effect sizes, brain outcome measures were normalized to z scores using the mean and SDs from the healthy control groups in both cross-sectional and longitudinal clinical studies.

#### Cross-sectional clinical study

Group comparisons of brain outcome measures were conducted using linear regression analysis, with the healthy control group serving as the reference category. The persistent and recovered PTSD groups were included as dummy variables within the model. Age and sex were included as covariates. In the PTSD group (combining persistent and recovered PTSD groups), linear regression analysis was used to examine the relationship between prefrontal GABA and CBF levels, adjusting for age and sex. To ensure the robustness of findings, sensitivity analyses were performed by excluding potentially influential observations identified through Cook’s distance.^24^

Mediation analyses^63^ explored whether prefrontal GABA levels influenced PTSD symptom severity through changes in prefrontal (mediation model 1) or limbic (mediation model 2) CBF levels. The significance of the mediation variable (M, representing either prefrontal or limbic CBF levels) was evaluated to determine its role in the causal pathway between the independent variable (X, prefrontal GABA levels) and dependent variable (Y, PTSD symptom severity). The total effect (*c*) of X on Y comprises the direct effect (*c’*) after accounting for the mediator and the indirect effect (*aXb*) through the M. A significant mediation effect was indicated by significant total (*c*) and indirect (*aXb*) effects, with a notable reduction in the direct effect (*c’*) upon the inclusion of M.^64^ Adjustments for age and sex was made in each model. A non-parametric bootstrapping method with 5,000 simulations was used to assess the indirect effects, considering an effect significant if the 95% confidence interval did not include zero.^65^

#### Longitudinal clinical study

Baseline between-group analyses were conducted using linear regression analysis, adjusting for age and sex. Within the trauma-exposed group, linear regression analysis explored the relationships between prefrontal GABA levels, PTSD symptom severity, and CBF levels, again adjusting for age and sex. Sensitivity analyses were conducted to evaluate the robustness of models by excluding potentially influential data points identified through Cook’s distance.^24^

Mixed-effects linear regression analysis assessed the effects of time on changes in CAPS total scores and specific brain outcome measures within the trauma-exposed group, with age and sex adjustments. Partial correlation analysis investigated the relationship between changes in these brain outcome measures and PTSD symptom severity, considering age and sex as covariates.

### Postmortem Human Brain Study

#### Human brain samples

Human brain samples from both normal subjects and patients with PTSD underwent neuropathological examination. These samples were prepared according to established protocols by the Boston University Alzheimer’s Disease Research Center (BUADRC) and the PTSD Brain Bank at the Jamaica Plain Veterans Administration. Informed consent for brain donation was obtained from the next-of-kin of all subjects. This study received an exemption from the Institutional Review Board of the Boston University School of Medicine, as it exclusively utilized postmortem tissue samples, which are not classified as human subjects under regulatory guidelines. All postmortem tissue samples used in this study were derived from male subjects due to limitations in available matched samples from the brain repository. Detailed sample information is provided in Supplementary Table 8.

### Double staining immunohistochemistry for the postmortem human brains

#### First staining

Paraffin-embedded tissues were sectioned coronally at a 20 μm thickness. Endogenous alkaline phosphatase activity was blocked by incubating with 3% hydrogen peroxide in Tris-buffered saline (TBS). Sections were blocked with 2.5% normal horse serum (Vector Laboratories) for 1 h, followed by 24 h of incubation with the following rabbit primary antibodies: anti-S100β (1:100, Abcam ab41548), anti-MAOB (1:100, Abcam ab175136, Santacruz sc-515354), and anti-ABAT (1:100, Abcam ab108249). Afterward, sections were washed three times with TBS and incubated with ImmPRESS-AP anti-rabbit IgG (alkaline phosphatase) polymer detection reagent (Vector Laboratories: MP-5402) for 30 min at room temperature. The development of signals for S100β, MAOB, and ABAT was achieved using the Vector red alkaline phosphatase substrate kit (Vector Laboratories).

#### Second staining

Subsequently, sections previously stained for S100β, MAOB, and ABAT were incubated with guinea pig anti-GABA (1:100, Millipore ab175) and chicken anti-GFAP (1:200, Abcam ab4647) antibodies for 24 h. After washing three times with TBS, slides were processed using the Vector ABC kit (Vector Laboratories, Inc., Burlingame, CA, USA). GABA and GFAP immunoreactivities were visualized using DAB chromogen (Thermo Fisher Scientific, Meridian, Rockford, IL, USA). Finally, the double-stained slides were dehydrated through a graded ethanol series (70%, 80%, and 95% [once], and 100% [twice]), cleared in xylene, and mounted.

### Animal studies

#### Animals

All mice were housed in groups within a controlled environment, maintaining consistent humidity and temperature, under a 12 hour light/dark cycle (8:00 AM–8:00 PM). They had unrestricted access to food and water. The Institutional Animal Care and Use Committee of the Institute for Basic Science (Daejeon, South Korea) approved all animal care and experimental procedures. Male C57BL/6 J mice aged 8 to 10 weeks were used. 6 to 7-week-old male C57BL/6 J mice for MAOB shRNA injections. Additionally, MAOB KO (129/SvImJ strain)^35^ and WT littermates, both male and female, on a C57BL/6 J background and aged 8 to 10 weeks, were used for the MAOB-OE model. Prior to experimentation, all mice underwent a 3-day habituation period in a novel chamber. Each experiment included age-matched control mice as the reference group, ensuring that separate sets of animals were employed for each specific assessment.

### Mouse models of PTSD

We developed a mouse model of PTSD using a double-stress paradigm that combines conditioned fear stress and subsequent single prolonged stress (SPS).^32^ Over five consecutive days (day 1–5), mice underwent fear conditioning, where they learned to associate a repeated unconditioned stimulus (a single electric foot shock per day; 1 mA, 3-second duration) with a specific chamber environment (Coulbourn Instruments, USA). On day 6, mice randomly assigned to the PTSD group were subjected to the SPS protocol, which consisted of three sequential stressors. First, mice were physically restrained in a restrain chamber for 2.5 h. Immediately afterward, they were subjected to forced swimming for 20 min in the transparent acrylic tank (25 cm × 15 cm x 30 cm) filled to two-thirds with 25 °C water. Following swimming, mice were allowed to recover in their home cages for 15 min. Subsequently, mice were placed in a novel transparent chamber containing a cotton pad saturated with diethyl ether. The chamber was sealed, and mice were exposed to ether vapor until they lost their righting reflex (when they could no longer flip themselves upright upon being placed on their back). Once this endpoint was reached, mice were immediately removed from the ether chamber and placed in a recovery cage under continuous observation until they regained consciousness. Following SPS, mice remained undisturbed in their home cages for 7 days before undergoing assessments.^32^

Control mice were placed into the same footshock chamber each day but did not receive any foot shocks; they were only exposed to the chamber context. Similarly, for the ether exposure control, mice were placed in a chamber identical in appearance to the ether chamber but did not receive ether exposure. These controls ensured that handling and environmental conditions were consistent without inducing stress.

### KDS2010 treatment for MAOB inhibition

To establish the PTSD + KDS2010 group, PTSD-like models were treated orally with KDS2010 (10 mg/kg/day) via drinking water ad libitum for nine consecutive days, starting on day 6 following completion of the double-stress paradigm. To ensure accurate dosing, the average daily water intake per mouse was measured over three consecutive days prior to KDS2010 administration. Based on this average intake, the KDS2010 concentration in the drinking water was adjusted to deliver an approximate dose of 10 mg/kg/day. After treatment initiation, water consumption was monitored for an additional three days to confirm that the KDS2010 administration did not alter drinking behavior. Control mice received normal drinking water without KDS2010 under the same conditions. KDS2010, a highly selective and reversible MAOB inhibitor, was provided by Dr. Ki Duk Park (Korea Institute of Science and Technology, South Korea).^36^

### Behavioral assessments: contextual fear conditioning and extinction test

Contextual fear conditioning, extinction, and retrieval sessions were conducted in the same shock chamber to evaluate PTSD-like behaviors in mice. PTSD-like mouse models were generated using a sequential foot shock protocol during the fear acquisition phase based on established methods^32^. Mice underwent fear conditioning over five consecutive days (days 1–5), during which freezing responses—quantified as the percentage of time spent freezing—were measured using Freezeframe 4 software (Actimetrics, IL, USA) to assess fear acquisition and consolidation. Each footshock was delivered at 1 mA for 3-s during the conditioning sessions. To ensure that KDS2010 treatment did not influence the acquisition phase, the compound was administered only after the SPS (single prolonged stress) protocol was completed, starting from day 6. Following a 1-week undisturbed period, mice were re-exposed to the shock chamber for a 25-min extinction session in the same context without foot shocks to assess extinction learning. The next day, mice underwent a retrieval session to evaluate fear memory recall, and freezing responses were measured again. To further validate the PTSD-like phenotype, we included a footshock-only group that underwent the same contextual fear conditioning protocol without subsequent SPS. This allowed us to isolate the effect of prolonged stress on fear extinction, demonstrating that PTSD-model mice (fear conditioning + SPS) exhibited impaired extinction retrieval compared to the footshock-only group, despite acquiring similar levels of fear during conditioning.

Throughout all sessions, the shock chamber and waste tray were thoroughly cleaned with 70% ethanol and water before and after each use to minimize olfactory cues. This design ensured consistency in behavioral testing while isolating the effects of KDS2010 on fear extinction and retrieval without interfering with initial fear acquisition.

#### Y-maze test

The Y-maze apparatus (15 cm high, 8 cm wide, and 40 cm long) consists of three identical arms angled at 120° from one another. Each mouse was placed in the start arm of the maze and allowed to explore for 5 min. Two measures, the number of entries into each arm and the percentage of alternation, were assessed to measure working memory performance. The number of entries into each arm and the sequence of entries were recorded with a camera. An entry was counted when all four limbs of a mouse were in the arm. Spontaneous alternation was defined as entries into three different arms sequentially (i.e., ABC, ACB, BAC, BCA, CAB, or CBA). Alternation was calculated with the following formula: Alternation (%) = number of spontaneous alternations / (total number of arm entries - 2) x 100.

### Genetic mouse models for MAOB inhibition and overexpression

We implemented procedures to establish the MAOB KD mouse model prior to the double-stress paradigm. Mice were deeply anesthetized with 1% isoflurane and placed in a stereotaxic frame (RWD Life Science, China). Bilateral craniotomies were performed above the prefrontal cortex at coordinates anterior-posterior (AP), +1.7 mm, and medial-lateral (ML), ±0.3 mm from the bregma). To achieve cell-type specific knockdown, we prepared a mixture of adeno-associated virus (AAV)-GFAP104-Cre-mCherry virus with either Lenti-pSico-scrambled-EGFP (control) or Lenti-pSico-MAOB*-*shRNA-EGFP (MAOB KD). Using a syringe pump (KD Scientific, MA, USA), we bilaterally microinjected 0.8 µl of virus mixture into either the IL (dorsoventral [DV], −3.2 mm from the bregma) or PL cortex (DV, −2.2 mm from the bregma) at a rate of 0.1 µl/min. The needle remained in place for an additional 10 min to facilitate diffusion, followed by careful withdrawal and surgical closure. After two weeks, to allow for in vivo gene silencing, we initiated the double-stress paradigm to establish the PTSD model. To overexpress astrocytic MAOB in the IL cortex of MAOB KO mice, we bilaterally microinjected AAV-GFAP104-MAOB-2A-EGFP or AAV-GFAP104-GFAP in the IL, following the same procedures described above. MAOB sequence was used as previously described.^19^

### Immunohistochemistry

For paraformaldehyde (PFA) fixation, mice were deeply anesthetized with isoflurane and perfused sequentially with heparinized normal saline, followed by 4% PFA and 0.5% glutaraldehyde. Extracted brains were post-fixed overnight in the same fixative at 4 °C, then cryoprotected in 30% sucrose solution for 48 h. Brains were subsequently embedded in the Optimal Cutting Temperature (OCT) compound and sectioned into 30-µm-thick coronal prefrontal slices using a CM1860 Cryostat (Leica, Germany). Sections were collected in 0.1 M phosphate-buffered saline (PBS) and blocked for 1.5 h at room temperature in a blocking solution containing 0.3% Triton X-100, 2% donkey serum, and the appropriate concentration of goat serum in 0.1 M PBS. This was followed by overnight incubation at 4 °C with a mixture of primary antibodies in the blocking solution with gentle shaking. Tissues were washed three times in 0.1 M PBS and incubated with secondary antibodies for 1.5 h. DAPI staining (1:1000; Pierce, WI, USA) was performed during the second wash step, followed by a final wash with 0.1 M PBS. Sections were mounted on saline-coated slides (Muto Pure Chemical 5116-20 F) using a fluorescent mounting medium (Dako) and imaged with an LSM900 confocal microscope (ZEISS, Germany). The primary antibodies used in the immunohistochemistry included: rabbit anti-S100β (1:1000, Abcam ab41548), mouse anti-S100β (1:500, Sigma S2532), guinea pig anti-GABA (1:200, Millipore ab175), rabbit anti-MAOB (1:200, Abcam ab175136), chicken anti-GFAP (1:1500, Abcam ab4647), rabbit anti-ABAT (1:500, Abcam ab108249), and rabbit anti-putrescine (1:200, MyBiosource MBS2090435). Fluorescent secondary antibodies included doneky anti-rabbit Alexa 405, donkey anti-rabbit Alexa 488, doneky anti-rabbit Alexa 594, donkey anti-mouse Alexa 594, donkey anti-chicken 647, donkey anti-gunieapig Alexa 594, and donkey anti-gunieapig Alexa 647 were sourced from Jackson ImmunoResearch Laboratories and diluted (1:500) in the blocking solution.

Confocal microscopic images of immunostained tissues were analyzed using ImageJ software (NIH, version 1.53c) and Imaris software (Oxford Instruments, version 9.0.1). To quantify fluorescence intensity, images of S100β and GFAP staining were converted to binary files, with ROIs defined to encompass astrocytes showing positive staining. Similarly, NeuN images were processed to create binary masks for isolating neuronal populations. Within these ROIs, the mean fluorescence intensity for GABA, MAOB, ABAT, and putrescine was measured. Additionally, the number of NeuN-positive DAPI signals within a 50 × 50 μm^2^ area of the PFC was quantified.

In the shRNA PTSD group, astrocyte-specific fluorescence intensities were measured in areas containing both DAPI and mCherry signals indicating Lenti-pSico-MAOB-shRNA-GFP (Sc shRNA) and AAV-GFAP104-Cre-mCherry expression. To minimize potential contamination from minor neuronal signals, an arbitrary boundary was defined around each astrocyte based on its characteristic morphology, including visible processes and branches.

S100β and GFAP-immunostained confocal images were stacked and maximally projected for the morphological analysis of astrocytes. Sholl analysis, performed with a plugin in ImageJ software (NIH, version 1.53c.), automatically drew concentric circles from the center of the DAPI signal outward to the ends of the astrocyte processes, analyzing the number of intercepts and the ramification index.

### Electrophysiological measurements

Tonic GABA and sIPSCs were recorded in the IL cortex of mice using brain slices prepared through the following procedures. Mice were deeply anesthetized with isoflurane and decapitated. Brains were rapidly extracted and immersed in chilled cutting solution (250 mM sucrose, 26 mM NaHCO_3_, 10 mM D(+)-glucose, 4 mM MgCl_2_, 3 mM myo-inositol, 2.5 mM KCl, 2 mM sodium pyruvate, 1.25 mM NaH_2_PO_4_, 0.5 mM ascorbic acid, 0.1 mM CaCl_2_, and 1 mM kynurenic acid, pH 7.4, continuously oxygenated with 95% O_2_ and 5% CO_2_). Coronal slices (300 µm) were prepared using a vibrating microtome (PRO7N; DSK, Japan) and transferred to a chamber containing oxygenated artificial cerebrospinal fluid (aCSF: 130 mM NaCl, 24 mM NaHCO_3_, 1.25 mM NaH_2_PO_4_, 3.5 mM KCl, 1.5 mM CaCl_2_, 1.5 mM MgCl_2_, and 10 mM D(+)-glucose, pH 7.4). Slices were incubated at room temperature for at least one hour before recordings.

For the recordings, slices were transferred to a recording chamber continuously perfused with aCSF and visualized on an upright microscope (Zeiss, Germany) using a 60x water immersion objective, infrared differential interference contrast optics, and a CMOS camera (Hamamatsu, Japan). Whole-cell patch-clamp recordings were obtained from pyramidal neurons within the IL cortex at a holding potential of −70 mV. Patch pipettes (4–7 MΩ resistance, Warner Instruments, MA, USA) were prepared using a micropipette puller (PC-100, Narishige, Japan) and filled with an internal solution containing: 135 mM CsCl, 4 mM NaCl, 0.5 mM CaCl_2_, 10 mM HEPES, 5 mM EGTA, 2 mM MgATP, 0.5 mM Na_2_GTP, 10 mM QX-314, and adjusted to pH 7.2 with CsOH, 278–285 mOsmol. Prior to measuring the tonic GABA current, the baseline current was stabilized in the presence of D-AP5 (50 µM) and CNQX (20 µM). The frequency and amplitude of sIPSCs were recorded at this stage. Tonic GABA current amplitude was determined using Clampfit software (ver.10.4.1.10) by measuring the baseline shift following the bicuculline (50 µM) application.

For the spike probability, synaptic response in the layer 5 IL cortical pyramidal neurons was evoked by 0.1 Hz stimulation of the layer 2/3 fibers (100 µs duration; 50–800 µA intensity) via a constant current isolation unit. Layer 2/3 fibers were stimulated by placing a tungsten bipolar electrode in the IL cortex. Evoked patch pipettes (4–7 MΩ resistance) were filled with an internal solution containing: 120 mM potassium gluconate, 10 mM KCl, 1 mM MgCl_2_, 0.5 mM EGTA, 40 mM HEPES, and adjusted to pH 7.2 with KOH). Data were acquired using a Digidata 1550B and an Axon Multiclamp 700B patch-clamp amplifier (Molecular Devices, CA, USA), utilizing pClamp software (version 11.0.3).

### CBF measurements

Mice were initially anesthetized with 3% isoflurane and maintained at 1.5% throughout the surgery. Body temperature was controlled at around 37 °C using a temperature-controlled heating pad system (FHC Inc., USA). Mice were placed in a stereotaxic frame (Kopf Instruments, USA), and a rectangular craniotomy was performed over the medial prefrontal cortex (AP, 1–3 mm and ML, 0–1.5 mm from the bregma) using a dental drill (Microtorque II, Ram Products, USA). The dura mater was carefully removed, and the exposed brain was kept moist with saline. Anesthesia was transitioned to urethane (1.25 g/kg, intraperitoneally) for CBF measurement. An LDF probe (PERIMED, Sweden) was positioned within the cingulate cortex (AP, +1.54 mm, ML, +0.36 mm, and dorsal-ventral [DV], −1.2 mm from the bregma). To evoke neuronal activation within the medial prefrontal cortex, a focal application of 1 mM NMDA was delivered to the cingulate cortex (AP, +2.04 mm, ML, +0.36 mm, and DV, −1.2 mm from the bregma) for 50 ms at 8 psi via a pressure-ejection system (Picospritzer II, Parker Hannifin, USA). To minimize direct drug and pressure effects, the NMDA-filled glass pipette was positioned 250 μm away from the LDF probe. LDF signals were sampled at 1 kHz by an LDF spectrometer (PeriFlux System 5000, PERIMED, Sweden) and digitally acquired through a Plexon system. Analysis of NMDA-evoked CBF changes included assessing the peak amplitude, time to peak, and area under the curve (AUC), with the AUC calculated from the onset of stimulation to 100 sec post-stimulation.

### Statistical analysis

Data presentation and statistical analyses for mouse models were conducted using GraphPad Prism version 10 (GraphPad Software, CA, USA).

A one-way ANOVA followed by Tukey’s multiple comparison test was used to compare the relevant outcome variables among the control, PTSD, and PTSD + KDS2010 groups. For the comparisons between the two groups, an independent t-test or Mann-Whitney U test was used depending on the data distribution. Animals were housed under identical conditions and randomly assigned to experimental groups to minimize bias. Importantly, groups designated to receive KDS2010 were randomized before PTSD modeling to ensure unbiased allocation, and group assignments were not determined based on experimental outcomes. For all analyses, a *P*-value < 0.05 was considered statistically significant. Data are presented as the mean ± standard error of the mean. To evaluate whether group differences in histological outcomes remained significant when accounting for subject- or animal-level variability, we reanalyzed all relevant datasets using linear mixed-effects models. In these models, the experimental group (Control vs. PTSD) was treated as a fixed effect, and subject or animal ID was modeled as a random intercept to account for intra-group clustering. In addition to statistical significance (P-values), we quantified the effect size of group differences using the coefficient of determination (R2), which represents the proportion of variance in the outcome variable explained by the fixed effect.

## Supplementary information

Supplementary material include Supplementary Figures and Tables

Clinical study protocol

Data S1 for RNA seq in Supplementary Fig. 3a

Raw source data1

Raw source data2

Raw source data3

Raw source data4

Raw source data5

Raw source data6

## Data Availability

The datasets generated in this study, including previously published RNA sequencing raw data, are available in the Mendeley Data repository (https://data.mendeley.com/) with DOI: 10.17632/5hd3f79d6m.1. The remaining data will be made available upon reasonable request, subject to the terms of consent and data use limitations for the subjects.
