# Supplementary material for: Astrocytic gamma-aminobutyric acid dysregulation as a therapeutic target for posttraumatic stress disorder
Source: Signal Transduct Target Ther. 2025 Jul 28;10:240. doi: 10.1038/s41392-025-02317-5 (PMC12301456; doi:10.1038/s41392-025-02317-5)
Supplement: Supplementary file 1 — Supplementary material include Supplementary Figures and Tables [file 41392_2025_2317_MOESM1_ESM.docx]

Supplementary Materials for

Astrocytic gamma-aminobutyric acid dysregulation as a therapeutic target for posttraumatic stress disorder

Sujung Yoon†, Woojin Won†, Suji Lee†, Kayoung Han, Eunji Ha, Juheon Lee, Seung Jae Hyeon, Yoonji Joo, Haejin Hong, Hyangwon Lee, Yumi Song, Ki Duk Park, Bertrand R. Huber, Junghee Lee, Richard A. E. Edden, Minah Suh, Hoon Ryu, C. Justin Lee*, In Kyoon Lyoo*

Correspondence to: cjl@ibs.re.kr / inkylyoo@ewha.ac.kr

**This PDF file includes:**

Materials and Methods

Figures S1 to S11

Tables S1 to S13

Materials and Methods

RNA-Seq Data Analysis and Visualization

Differentially expressed genes (DEGs) in astrocytes were analyzed using a previously published single-cell RNA-sequencing dataset from PTSD and negative control cohorts (Daskalakis et al., Science, 2024, DOI: 10.1126/science.adh3707)^1^. Specifically, data were extracted from Supplementary Table 2 of the study, focusing on S2a-3, which contains RNA gene expression profiles from the medial prefrontal cortex (mPFC) of PTSD subjects. From the dataset, a total of 1500 single-cell gene lists were examined, and genes related to astrogliosis were highlighted, including markers such as GFAP, MAOB, and ALDH family members. Differential transcriptomic analysis was performed to identify significantly upregulated and downregulated genes between PTSD and control groups. Visualization was achieved by constructing a volcano plot using GraphPad Prism 10 and R (v4.1.2). Genes with a log2 fold change ≥ 1 and adjusted p-value ≤ 0.05 were considered statistically significant. Astrocyte-specific DEGs were annotated based on prior functional classifications, emphasizing genes associated with astrocytic reactivity and metabolic pathways. The list of related genes analyzed in this study is provided in the Data S1 as part of the supplementary materials.

Open field test

Animals were placed in a 40 cm × 40 cm chamber with no markings and allowed to explore uninterrupted for 10 min. They were tracked for the last 8 min of the recording (Ethovision XT, Noldus) to measure the total distance travelled and the velocity.

Dopamine imaging in striatal slices using GRABDA2m sensors

To evaluate evoked dopamine release in the striatum, mice were deeply anesthetized with 1-2% isoflurane and decapitated. Brains were rapidly extracted and submerged in ice-cold dissection buffer (in mM): 212.5 sucrose, 26 NaHCO3, 10 D-glucose, 5 MgCl2, 3 KCl, 1.25 NaH2PO4, and 0.1 CaCl2; pH 7.4, saturated with 95% O2 and 5% CO2. Coronal slices (300 μm) containing the dorsal striatum were prepared using a vibratome (LinearSlicer Pro7, Dosaka EM Co., Ltd) and incubated for at least 1 hour in artificial cerebrospinal fluid (aCSF; in mM: 130 NaCl, 24 NaHCO3, 3.5 KCl, 1.25 NaH2PO4, 1.5 CaCl2, 1.5 MgCl2, and 10 glucose; pH 7.4) at room temperature.

After stabilization, slices were transferred to a perfusion chamber with continuous aCSF flow (1-2 mL/min). GRABDA2m fluorescence was visualized under an upright fluorescence microscope (Zeiss Axioscope) using a 10x objective and blue LED illumination (CoolLED). Phasic dopamine release was evoked by delivering a single electrical pulse (100 μA, 2 ms, 1 pulse) via a bipolar electrode placed near the sensor-expressing region. Evoked fluorescence responses (ΔF/F₀) were measured at ~10 manually selected regions of interest (ROIs) per slice.

To normalize evoked responses across slices and animals, a bath application of 20 μM dopamine was subsequently applied to induce maximal GRABDA2m activation. Evoked dopamine responses were expressed as a percentage of the maximal 20 μM dopamine-induced fluorescence (% of maximum DA response). Fluorescence data acquisition and ROI analysis were performed using Imaging Workbench (INDEC BioSystems) and ImageJ (NIH, version 1.53c).

**References for Supplementary Text**

1. Daskalakis, N. P. et al. Systems biology dissection of PTSD and MDD across brain regions, cell types, and blood. *Science*. **384**, eadh3707, (2024).


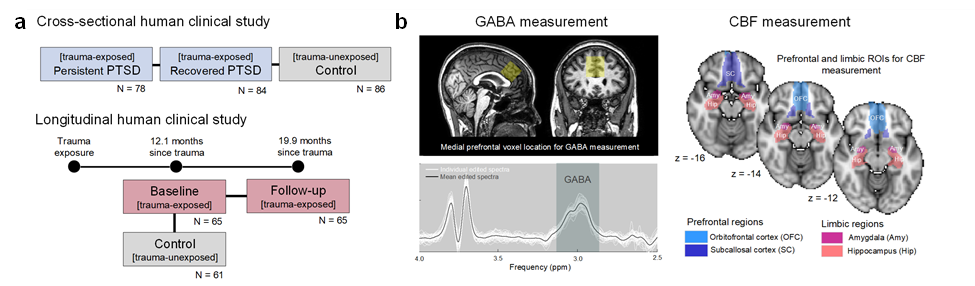
Figure S1.

**Study design and outcome measures of human brain studies (Related to Figures 2 and 3). a** Study designs and participants in the cross-sectional and longitudinal human clinical studies. The study participants in both studies did not overlap. Cross-sectional clinical study included trauma-exposed individuals who had recovered from PTSD (N = 84, the recovered PTSD group), and trauma-exposed individuals with current and persistent PTSD (N = 78, the persistent PTSD group), and healthy individuals (N = 86, the healthy control group). Prefrontal GABA levels and CBF levels in the prefrontal and limbic areas were measured in each study participant. Longitudinal clinical study involves an independent human sample of trauma-exposed individuals (N = 65, the trauma-exposed group) and healthy individuals (N = 61, the healthy control group). For the trauma-exposed group, brain outcome measures (prefrontal GABA levels and CBF levels in the prefrontal and limbic areas) were assessed at baseline (12.1 months since trauma exposure) and at a follow-up approximately 8 months later (19.9 months since trauma exposure). **b** Brain outcome measures included GABA levels in the prefrontal VOI, assessed using ^1^H-MRS, and CBF levels in the prefrontal and limbic ROIs, measured using ASL perfusion MRI.

Abbreviations: PTSD, posttraumatic stress disorder; GABA, gamma-aminobutyric acid; ROI, region-of-interest; VOI, voxel-of-interest; CBF, normalized resting-state cerebral blood flow; ^1^H-MRS, proton magnetic resonance spectroscopy; ASL, arterial spin labeling; MRI, magnetic resonance imaging.


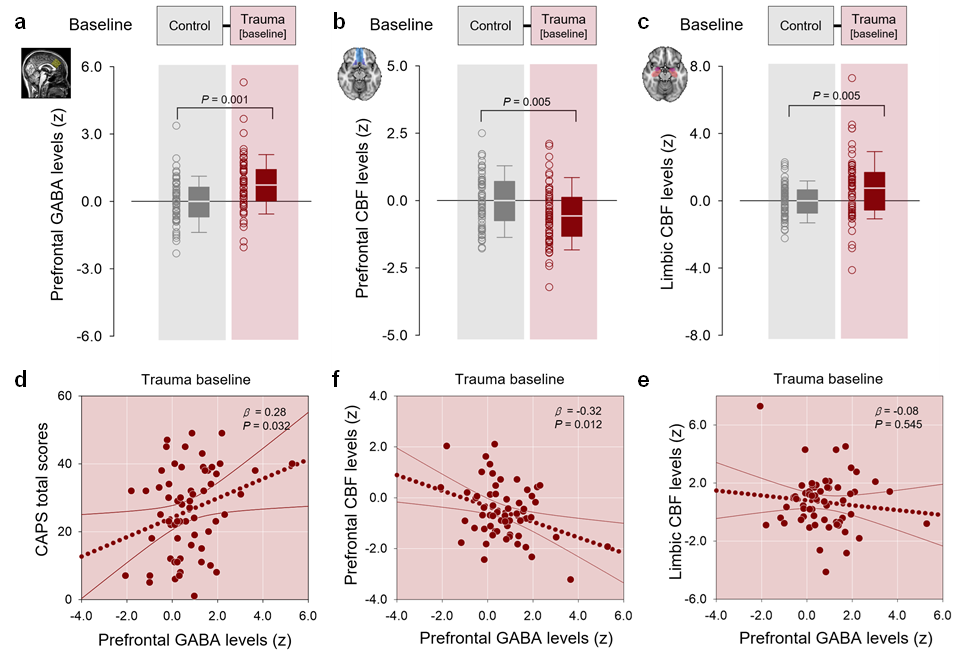


Figure S2.

**Results from the baseline assessments in the longitudinal clinical study (Related to Figure 3). a-c** The trauma-exposed group showed higher prefrontal GABA levels (*β* = 0.28, *P* = 0.001) (**a**), lower prefrontal CBF levels (*β* = -0.24, *P* = 0.005) (**b**), and higher limbic CBF levels (*β* = 0.25, *P* = 0.005) (**c**), than the healthy control group. (d) Higher prefrontal GABA levels were associated with more severe PTSD symptoms (*β* = 0.28, *P* = 0.032). **e** A significant negative relationship was observed between the prefrontal GABA levels and prefrontal CBF levels (*β* = -0.32, *P* = 0.012). **f** However, the prefrontal GABA level had no significant relationship with the limbic CBF level (*β* = -0.08, *P* = 0.545). Error bars in the graphs indicate standard errors of the mean.

Abbreviations: GABA, gamma-aminobutyric acid; CBF, normalized cerebral blood flow; CAPS, Clinician-Administered Posttraumatic Stress Disorder Scale for DSM-5.


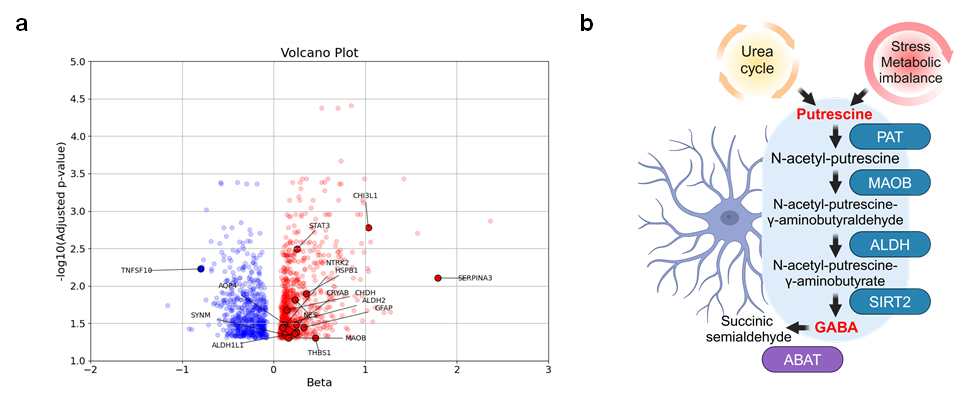


Figure S3.

**Schematic image of the enzyme pathway for GABA synthesis and degradation. a** Volcano plot depicting differentially expressed genes (DEGs) in astrocytes from a previously published PTSD single-cell RNA-sequencing database. Significantly upregulated and downregulated genes are highlighted. Notably, increased expression of GFAP, MAOB, and members of the ALDH family is observed, indicating astrocytic reactivity and metabolic alterations associated with PTSD (*Daskalakis et al., Science, 2024*). **b** Astrocytic GABA is known to be synthesized by diverse enzymes, including MAOB, and degraded by catabolic enzymes such as ABAT. Putrescine is possibly dysregulated by stress metabolic imbalance or urea cycle in astrocytes. MAOB uses putrescine as a pre-substrate polyamine molecule in the astrocytes. Abbreviations: GABA, gamma-aminobutyric acid; PAT, putrescine aminotransferase; MAOB, monoamine oxidase B; ALDH, Aldehyde dehydrogenase family; SIRT2, Sirtuin2; ABAT, 4-aminobutyrate aminotransferase. Figure created with BioRender.com.


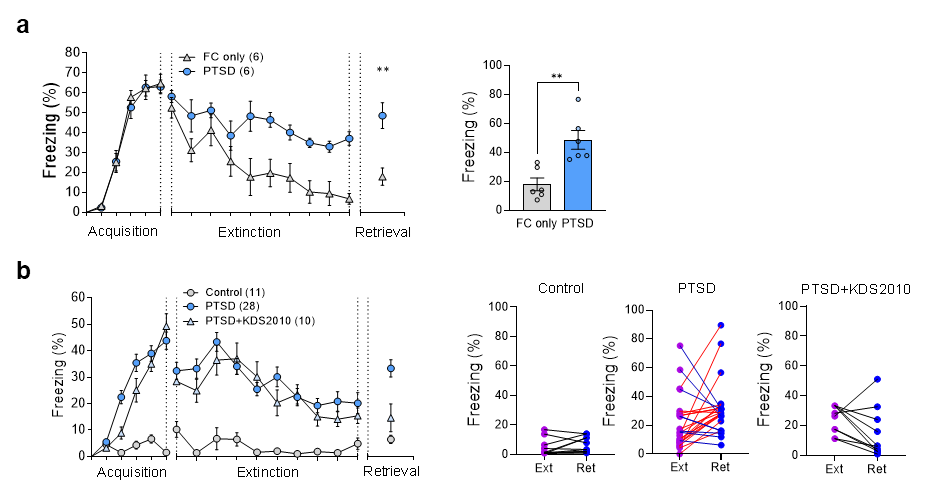


Figure S4.

**Validation of impaired fear extinction and retrieval in the PTSD-like mouse model.** **a** Behavioral data from the contextual fear conditioning, extinction, and extinction retrieval in the footshock-only and PTSD-like mouse. Summary graph of freezing percentage during the retrieval session. **b** Behavioral data from the contextual fear conditioning, extinction, and extinction retrieval in the control, PTSD-like mouse, and PTSD-like mouse+KDS2010 groups (left). Paired comparison of freezing percentages during extinction and retrieval sessions in individual mice from the control, PTSD-like mouse, and PTSD-like mouse+KDS2010 groups. Error bars in the graphs indicate standard errors of the mean. ** P < 0.01. Abbreviations: PTSD, post-traumatic stress disorder; Ext, extinction; Ret, retrieval.


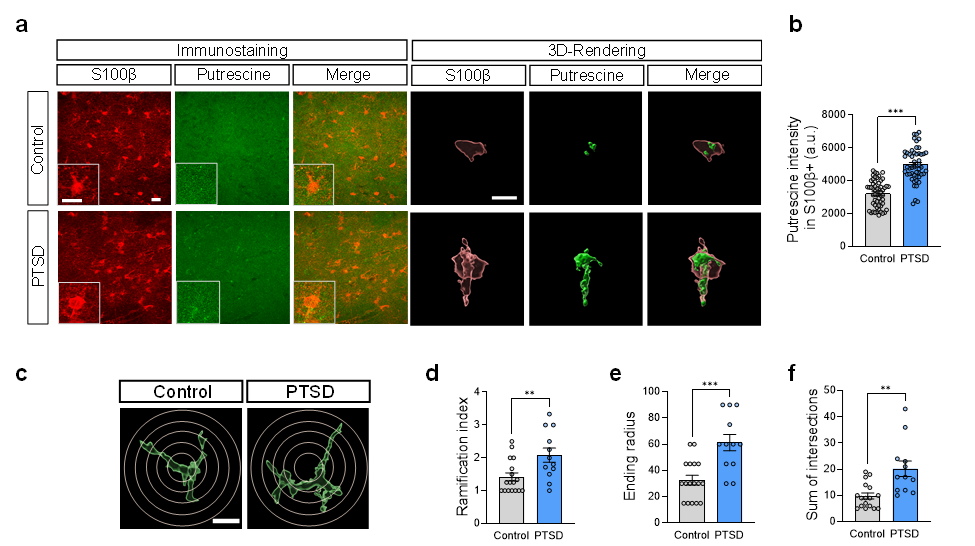


Figure S5.

**Increase of putrescine levels in PTSD mouse model. a** Representative confocal and Imaris images show putrescine and S100β-positive astrocytes in the control and PTSD groups (arrows indicate the inset). **b** Quantification of putrescine intensity in the S100β-positive astrocytic area. **c** Representative Sholl analysis images of GFAP in control and PTSD groups (scale bar, 10 μm). Related to Fig. 5h. **d-f** The summary graph shows the ramification index (d), ending radius (e), and sum of intersections (f) from GFAP-positive astrocytes. Error bars in the graphs indicate standard errors of the mean. ** P < 0.01; *** P < 0.001. Abbreviations: PTSD, post-traumatic stress disorder; S100β, S100 calcium-binding protein B; a.u., arbitrary unit.


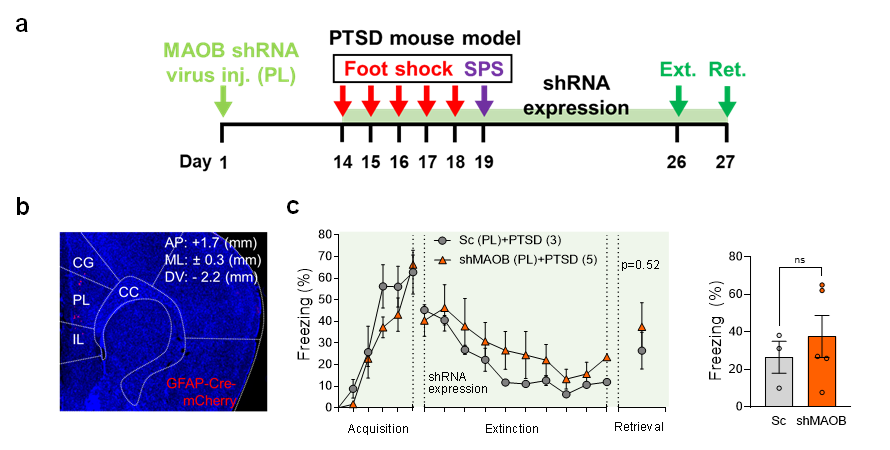


Figure S6.

**Results from the genetic mouse model to block astrocytic MAOB in the prelimbic (PL) cortex. a** Timeline of the shMAOB+PTSD (PL) and Sc+PTSD (PL) mouse models, fear extinction, and retrieval for behavioral assessment. **b** Astrocyte-specific gene-silencing of MAOB in the PL cortex. Specifically, a mixture of Lenti-pSico-MAOB-shRNA-EGFP (scrambled shRNA) and AAV-GFAP104-Cre-mCherry virus were bilaterally infused into the PL cortex (AP: +1.7 mm, ML: ± 0.3 mm, DV: -2.2 mm). **c** Behavioral data across contextual fear conditioning, extinction and the following extinction retrieval session in the Sc+PTSD (n = 3) and shMAOB+PTSD (n = 5) groups. Freezing behavior during the retrieval session did not differ between the groups (t = 0.69, P = 0.52). Error bars in the graphs indicate standard errors of the mean. Error bars in the graphs indicate standard errors of the mean. ns, not significant. Abbreviations: AAV, adeno-associated virus; Lenti, lentivirus; IL, infralimbic; PL, prelimbic; CG, anterior cingulate; CC, corpus callosum; MAOB, monoamine oxidase B; KD, knockdown; PTSD, posttraumatic stress disorder; shRNA, single hairpin RNA; SPS, single prolonged stress; AP, anterior-posterior; ML, medial-lateral; DV, dorsal-ventral; shMAOB, MAOB shRNA.


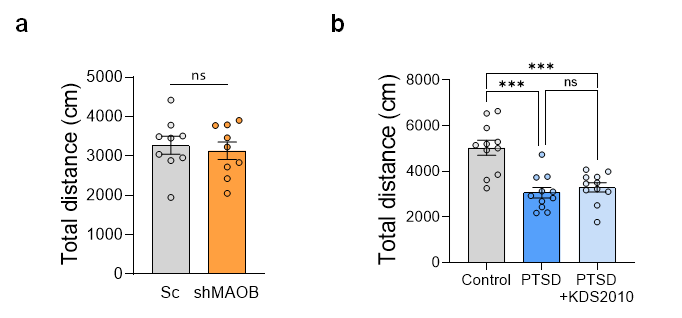


Figure S7.

**KDS2010 treatment and astrocytic MAOB knockdown do not induce nonspecific hyperlocomotor effects. a** Total distance traveled in the open field test in PTSD-like mice receiving scramble or astrocytic shMAOB viral constructs. No significant difference was observed between groups. These results confirm that the behavioral improvements induced by KDS2010 and astrocytic MAOB knockdown are not attributable to nonspecific increases in locomotor activity. **b** Total distance traveled in the open field test across control, PTSD-like, and PTSD+KDS2010-treated mice. PTSD-like mice exhibited a significant reduction in total distance compared to control mice, while KDS2010 treatment did not significantly alter locomotor activity relative to the PTSD group. Error bars in the graphs indicate standard errors of the mean. *** P < 0.001; ns, not significant. statistical analyses were performed using one-way ANOVA with Tukey’s post hoc test (a) and unpaired two-tailed t-test (b).Abbreviations: PTSD, post-traumatic stress disorder; Sc, scramble control; shMAOB, short hairpin RNA against MAOB.


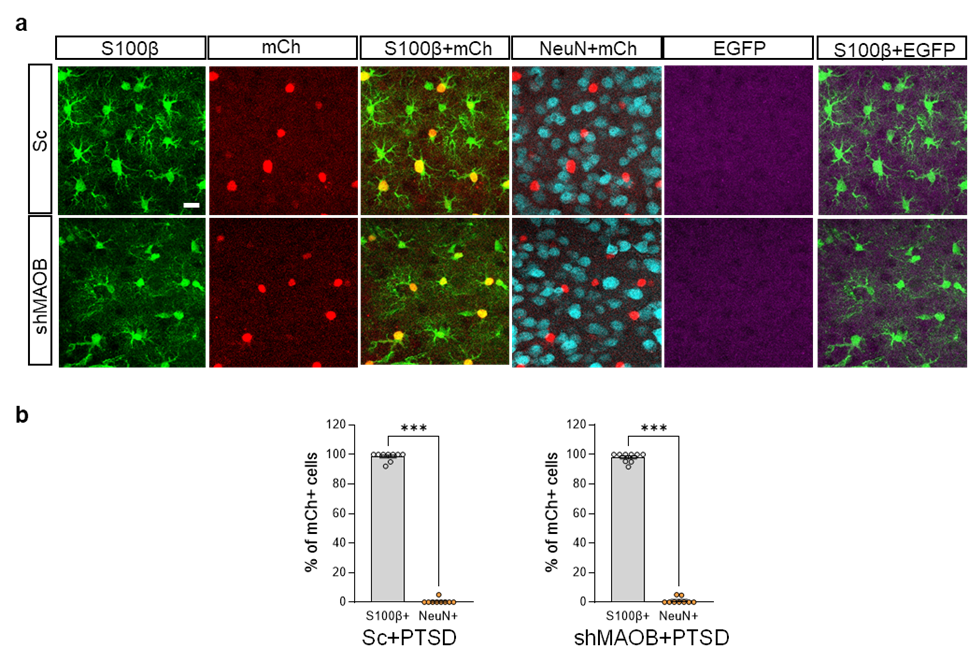
Figure S8.

**Validation of astrocyte-specific targeting of MAOB knockdown in the PTSD-like mouse model. a** Representative confocal image showing co-labeling of astrocytes (S100β, green), neurons (NeuN, cyan), mCherry (mCh, red), and EGFP (purple) in the infralimbic cortex of Sc+PTSD and shMAOB+PTSD groups. mCherry expression was largely confined to S100β-positive astrocytes with minimal overlap with NeuN-positive neurons, confirming astrocyte-specific viral targeting. EGFP signal was excised after Cre recombination, supporting successful knockdown (Scale bar, 20 μm). Related to Fig. 6f. **b** Quantification of the proportion of mCherry-positive cells that were S100β-positive (astrocytic) versus NeuN-positive (neuronal) in Sc+PTSD and shMAOB+PTSD groups. In the Sc+PTSD group, 98.6 ± 0.99% of mCherry-positive cells were S100β-positive and 0.6 ± 0.56% were NeuN-positive (n = 9 per group). In the shMAOB+PTSD group, 98 ± 1.1% of mCherry-positive cells were S100β-positive and 1.1 ± 0.7% were NeuN-positive (n = 9 per group). Over 85% of mCherry-positive cells colocalized with S100β, while less than 1% colocalized with NeuN, confirming the astrocyte-specific expression of the shMAOB vector. Error bars in the graphs indicate standard errors of the mean. *** P < 0.001. Abbreviations: PTSD, post-traumatic stress disorder; S100β, S100 calcium-binding protein B; NeuN, neuronal nuclei marker; mCh, mCherry; EGFP, enhanced green fluorescent protein.


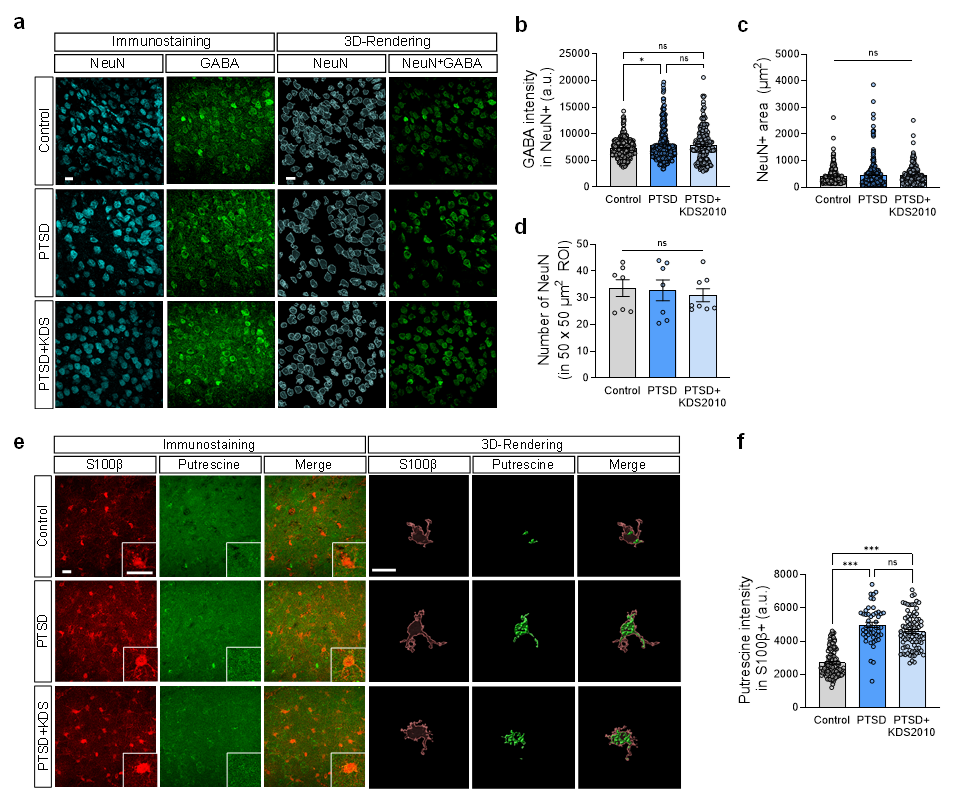


Figure S9.

**Neuronal GABA and astrocytic putrescine in PTSD-like mouse model.**

**a** Representative confocal and Imaris images show NeuN and GABA in the control, PTSD, and PTSD+KDS2010 groups. **b** Quantification of GABA intensity in the NeuN-positive area. **c,d** Quantification of NeuN-positive area (**c**) and number of NeuN in 50 x 50 μm^2^ area (**d**)**. e** Representative confocal and Imaris images show putrescine and S100β-positive astrocytes in the control, PTSD, and PTSD+KDS2010 groups. **f** Quantification of putrescine intensity in the S100β-positive astrocytic area. Error bars in the graphs indicate standard errors of the mean. * P < 0.05; *** P < 0.001; ns, not significant.


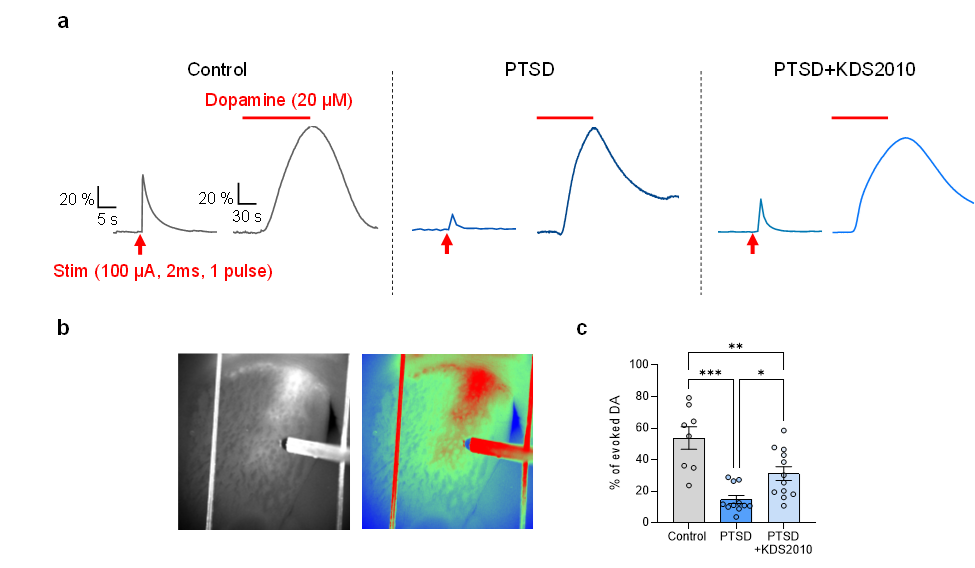


Figure S10.

**Ex vivo imaging of dopamine release in the striatum using GRABDA2m sensors.**

**a** Representative fluorescence traces showing evoked dopamine release in the control, PTSD-like, and PTSD+KDS2010-treated groups. A single electrical stimulation (100 μA, 2 ms, 1 pulse; red arrow) was delivered, and fluorescence changes were normalized by a subsequent bath application of 20 μM dopamine (red bar) to control for viral expression variability. **b** Representative images showing GRABDA2m expression in the striatum. Left: brightfield image indicating the optical fiber placement; right: pseudocolor fluorescence image confirming GRABDA2m expression. **c** Quantification of evoked dopamine responses normalized to the maximal fluorescence induced by 20 μM dopamine application. PTSD-like mice exhibited a significant reduction in evoked dopamine responses compared to controls, which was significantly rescued by KDS2010 treatment

Error bars in the graphs indicate standard errors of the mean. *P < 0.05; **P < 0.01; ***P < 0.001; ns, not significant. Abbreviations: PTSD, post-traumatic stress disorder; DA, dopamine; a.u., arbitrary unit.

Figure S11.

**Schematic overview of the study results.** Abbreviations: PTSD, posttraumatic stress disorder; GABA, a gamma-aminobutyric acid; CBF, normalized cerebral blood flow; MAOB, monoamine oxidase B; ABAT, 4-aminobutyrate aminotransferase.

|  | Healthy control group  (N = 86) | Persistent PTSD group (N = 78) | Recovered PTSD group (N = 84) | Statistical values | *P* |
| --- | --- | --- | --- | --- | --- |
| **Demographic characteristics** |  |  |  |  |  |
| Age, mean (SD), years | 41.3 (14.1) | 40.6 (13.0) | 40.8 (13.9) | *F* = 0.06 | 0.94 |
| Women sex, No. (%) | 75 (87.2) | 72 (92.3) | 74 (88.1) | *χ^2^* = 1.23 | 0.54 |
| Right handedness, No. (%) | 78 (90.7) | 71 (91.0) | 78 (92.9) | *χ^2^* = 0.29 | 0.86 |
| **Trauma exposure-related characteristics** |  |  |  |  |  |
| Age at trauma, mean (SD), years | NA | 38.7 (13.0) | 38.2 (14.0) | *t* = -0.21 | 0.84 |
| Time since trauma, mean (SD), months | NA | 24.0 (50.0) | 32.2 (43.0) | *t* = 1.13 | 0.26 |
| Type of exposed trauma, No. (%) |  |  |  |  |  |
| Physical violence | NA | 20 (25.6) | 26 (31.0) | *χ^2^* = 1.13 | 0.57 |
| Sexual/ interpersonal violence | NA | 50 (64.1) | 47 (56.0) |  |  |
| Life-threatening accidents or others | NA | 8 (10.3) | 11 (13.1) |  |  |
| CAPS total scores, lifetime, mean (SD) | NA | 43.7 (11.9) | 31.0 (12.6) | *t* = -6.57 | < 0.001 |
| CAPS total scores, current, mean (SD) | NA | 35.5 (9.8) | 10.4 (6.7) | *t* = -19.1 | < 0.001 |
|  | | | | | |

Table S1. Demographic and clinical characteristics of study participants in the cross-sectional clinical study
Demographic and clinical characteristics across group were examined using analysis of variance, independent-samples t-tests or chi-square tests, as appropriate. The demographic characteristics did not differ significantly among the healthy control, persistent PTSD, and recovered PTSD groups. Among the trauma exposure–related characteristics, there were no significant differences in age at trauma, time since trauma, or type of exposed trauma between the persistent and recovered PTSD groups. As expected, the persistent PTSD group showed higher total current and lifetime CAPS scores than the recovered PTSD group. ^1^H-MRS data for the healthy control group, persistent PTSD group, and recovered PTSD group were available for 85, 76, and 84 subjects, respectively. ASL data for the healthy control group, persistent PTSD group, and recovered PTSD group were available for 83, 76, 83 subjects, respectively. Abbreviations: PTSD, posttraumatic stress disorder; CAPS, Clinician Administered PTSD Scale for Diagnostic and Statistical Manual of Mental Disorders, fifth edition; SD, standard deviation; No, number; NA, not applicable; ^1^H-MRS, proton magnetic resonance spectroscopy; ASL, arterial spin labeling.

|  | Healthy control group  (N = 61) | Trauma-exposed group  (N = 65) | Statistical values | *P* |
| --- | --- | --- | --- | --- |
| **Demographic characteristics** |  |  |  |  |
| Age, mean (SD), years | 41.3 (13.3) | 40.2 (12.8) | *t* = 0.45 | 0.65 |
| Women sex, No. (%) | 40 (65.6) | 48 (73.9) | *χ^2^* = 1.02 | 0.31 |
| Right handedness, No. (%) | 59 (96.7) | 61 (93.9) | Fisher's exact *P* = 0.68 | |
| **Trauma exposure-related characteristics** |  |  |  |  |
| Age at trauma, mean (SD), years | NA | 39.2 (12.5) | NA | NA |
| Time since trauma, mean (SD), months | NA | 12.1 (15.6) | NA | NA |
| Type of exposed trauma, No. (%) |  |  |  |  |
| Physical violence | NA | 17 (26.2) | NA | NA |
| Sexual/ interpersonal violence | NA | 30 (46.2) |  |  |
| Life-threatening accidents or others | NA | 18 (27.7) |  |  |
| CAPS total scores, lifetime, mean (SD) | NA | 39.0 (13.7) | NA | NA |
| CAPS total scores at time 1 assessment, mean (SD) | NA | 26.3 (12.7) | NA | NA |
| CAPS total scores at time 2 assessment, mean (SD) | NA | 18.6 (13.7) | NA | NA |

Table S2. Demographic and clinical characteristics of study participants in the longitudinal human clinical study
Demographic and clinical characteristics across group were examined using independent-samples t-tests, chi-square tests, or Fisher's exact test, as appropriate. The demographic characteristics did not differ significantly between the trauma-exposed group and the healthy control group. Among 126 subjects, data from 59 subjects in the healthy control group and data from 62 subjects at baseline and follow-up in the trauma-exposed group were available for ^1^H-MRS analysis. For ASL analysis, data from 61 subjects in the healthy control group and data from 64 subjects at baseline and follow-up in the trauma-exposed group were available. Abbreviations: PTSD, posttraumatic stress disorder; CAPS, Clinician Administered PTSD Scale for Diagnostic and Statistical Manual of Mental Disorders, fifth edition; SD, standard deviation; No, number; NA, not applicable; ^1^H-MRS, proton magnetic resonance spectroscopy; ASL, arterial spin labeling.

| Variables | Relevant figure | Control (z) | | Persistent PTSD | | Recovered PTSD | |  | Control vs.  Persistent PTSD | | Control vs.  Recovered PTSD | |
| --- | --- | --- | --- | --- | --- | --- | --- | --- | --- | --- | --- | --- |
|  |  | N | mean (SEM) | N | mean (SEM) | N | mean (SEM) |  | *β* | *P* | *β* | *P* |
| Prefrontal GABA levels (z) | Fig. 2b | 85 | 0.000 (0.108) | 76 | 0.561 (0.172) | 84 | -0.068 (0.123) |  | 0.204 | 0.005 | -0.027 | 0.709 |
| Prefrontal CBF levels (z) | Fig. 2c | 83 | 0.000 (0.109) | 76 | -0.600 (0.131) | 83 | -0.044 (0.121) |  | -0.245 | 0.001 | -0.022 | 0.755 |
| Limbic CBF levels (z) | Fig. 2d | 83 | 0.000 (0.109) | 76 | 0.385 (0.135) | 83 | 0.624 (0.165) |  | 0.154 | 0.028 | 0.231 | 0.001 |

**Table S3. Detailed information about statistical values from the cross-sectional clinical study: Group comparisons**

Outcome variables were standardized to z scores using the mean and standard deviation (SD) of the control group. Linear regression analysis was conducted to compare outcome measures across groups. The healthy control group served as the reference category, while the persistent PTSD and recovered PTSD groups were included as dummy variables. Age and sex were included as covariates in the model.

Abbreviations: PTSD, posttraumatic stress disorder; SEM, standard error of mean; GABA, gamma-aminobutyric acid; CBF, normalized resting-state cerebral blood flow.

| Variables | Relevant figure | N | β | *P* |
| --- | --- | --- | --- | --- |
| Prefrontal CBF levels | Fig. 2e | 157 | -0.300 | < 0.001 |
| Limbic CBF levels | Fig. 2f | 157 | -0.085 | 0.278 |

**Table S4. Relationship of prefrontal GABA levels with prefrontal CBF and limbic CBF levels in the cross-sectional human clinical study**Linear regression analysis was conducted to examine the relationship between prefrontal GABA levels and CBF levels, with age and sex included as covariates in the model. Abbreviations: GABA, gamma-aminobutyric acid; CBF, normalized resting-state cerebral blood flow.

| Outcome variables | Relevant figure | Control group | | Trauma-exposed group | | *β* | *P* |
| --- | --- | --- | --- | --- | --- | --- | --- |
|  |  | N | mean (SEM) | N | mean (SEM) |  |  |
| Prefrontal GABA levels (z) | Fig. S2a | 59 | 0.000 (0.129) | 62 | 0.703 (0.156) | 0.279 | 0.001 |
| Prefrontal CBF levels (z) | Fig. S2b | 61 | 0.000 (0.127) | 64 | -0.560 (0.132) | -0.244 | 0.005 |
| Limbic CBF levels (z) | Fig. S2c | 61 | 0.000 (0.127) | 64 | 0.734 (0.231) | 0.249 | 0.005 |

**Table S5. Detailed information about statistical values from the longitudinal clinical study: Baseline assessments**Outcome variables were standardized to z scores using the mean and standard deviation (SD) of the control group. Linear regression analysis was conducted to compare outcome measures between groups after adjusting for age and sex. Abbreviations: SEM, standard error of mean; GABA, gamma-aminobutyric acid; CBF, normalized resting-state cerebral blood flow.

| Outcome variables | Relevant figure | N | *β* | *P* |
| --- | --- | --- | --- | --- |
| CAPS scores | Fig. S2d | 62 | 0.282 | 0.032 |
| Prefrontal CBF levels | Fig. S2e | 61 | -0.322 | 0.012 |
| Limbic CBF levels | Fig. S2f | 61 | -0.078 | 0.545 |
|  | | | | |

**Table S6. Relationship of prefrontal GABA levels with CAPS scores, prefrontal CBF, and limbic CBF levels at baseline in the longitudinal clinical study**

Linear regression analysis was conducted within the trauma-exposed group to examine the relationships between prefrontal GABA levels and each of the following: CAPS scores, prefrontal CBF, and limbic CBF level. Age and sex were included as covariates in all models. Abbreviations: GABA, gamma-aminobutyric acid; CBF, normalized resting-state cerebral blood flow.

| Outcome variables | Relevant figure | Baseline | | Follow-up | | *z* | *P* |
| --- | --- | --- | --- | --- | --- | --- | --- |
|  |  | N | mean (SEM) | N | mean (SEM) |  |  |
| CAPS scores | Fig. 3a | 65 | 26.3 (1.6) | 65 | 18.6 (1.7) | -5.87 | < 0.001 |
| Prefrontal GABA levels (z) | Fig. 3b | 62 | 0.703 (0.156) | 62 | 0.273 (0.149) | -2.43 | 0.015 |
| Prefrontal CBF levels (z) | Fig. 3c | 64 | -0.560 (0.132) | 64 | -0.372 (0.144) | 2.23 | 0.026 |
| Limbic CBF levels (z) | Fig. 3d | 64 | 0.734 (0.231) | 64 | 0.418 (0.161) | -1.86 | 0.062 |
|  | | | | | | | |

**Table S7. Detailed information about statistical values from the longitudinal clinical study: Follow-up assessments**

Mixed-effects linear regression analysis was performed within the trauma-exposed group to examine the effects of time on changes in CAPS scores, prefrontal GABA levels, prefrontal CBF levels, and limbic CBF levels. Age and sex were included as covariates in the models. Abbreviations: SEM, standard error of mean; CAPS, Clinician Administered PTSD Scale for Diagnostic and Statistical Manual of Mental Disorders, fifth edition; GABA, gamma-aminobutyric acid; CBF, normalized resting-state cerebral blood flow.

| Number | Case | Age | Sex | Race | Cause of Death |
| --- | --- | --- | --- | --- | --- |
| 1 | Normal | 75 | Male | Caucasian | Cardiac Arrest |
| 2 | Normal | 68 | Male | African American | Cardiac Arrest |
| 3 | Normal | 67 | Male | Hispanic | Heart Failure |
| 4 | Normal | 59 | Male | Hispanic | Cardiopulmonary Failure |
| 5 | Normal | 46 | Male | Hispanic | Cardiac Arrest |
| 1 | PTSD | 31 | Male | Caucasian | Suicide |
| 2 | PTSD | 54 | Male | African American | Cardiopulmonary Failure |
| 3 | PTSD | 65 | Male | Caucasian | Cardiorespiratory Arrest |
| 4 | PTSD | 71 | Male | Caucasian | Suicide |
| 5 | PTSD | 38 | Male | Caucasian | Suicide |

**Table S8. Information about postmortem brain tissue samples from normal subjects and patients with PTSD in postmortem human brain study**

| Outcome variables | Relevant figure | Control | | PTSD | | *t* | *P* | *R^2^* |
| --- | --- | --- | --- | --- | --- | --- | --- | --- |
|  |  | n | mean (SEM) | n | mean (SEM) |  |  |  |
| GABA in S100β+ area, fold | Fig. 4c | 30 | 1.00 (0.06) | 30 | 3.93 (0.07) | 31.58 | < 0.001 | 0.9452 |
| MAOB in GFAP+ area, fold | Fig. 4f | 30 | 1.00 (0.04) | 30 | 1.78 (0.03) | 15.47 | < 0.001 | 0.8407 |
| ABAT in GFAP+ area, fold | Fig. 4i | 30 | 1.00 (0.04) | 30 | 0.55 (0.01) | 10.95 | < 0.001 | 0.8003 |
| S100β+ area, fold | Fig. 4j | 30 | 1.00 (0.07) | 30 | 3.29 (0.20) | 10.74 | < 0.001 | 0.7596 |
| GFAP+ area, fold | Fig. 4k | 30 | 1.00 (0.04) | 30 | 2.00 (0.11) | 8.48 | < 0.001 | 0.6684 |
| Sum of intersections | Fig. 4m | 20 | 24.1 (1.0) | 20 | 44.3 (1.3) | 12.68 | < 0.001 | 0.8189 |

Table S9. Detailed information about statistical values from postmortem human brain study
The independent t-test was used to compare outcome variables between the control and PTSD groups. Abbreviations: PTSD, posttraumatic stress disorder; SEM, standard error of the mean; GABA, gamma-aminobutyric acid; S100β, S100 calcium-binding protein B; MAOB, monoamine oxidase B; GFAP, glial fibrillary acidic protein; ABAT, 4-aminobutyrate aminotransferase.

| Outcome variables | Relevant figure | Control | | PTSD | | *t* | *P* | *R^2^* |
| --- | --- | --- | --- | --- | --- | --- | --- | --- |
|  |  | n | mean (SEM) | n | mean (SEM) |  |  |  |
| *Immunohistochemistry* |  |  |  |  |  |  |  |  |
| GABA in S100β+ area, a.u. | Fig. 5f | 50 | 122.7 (6.2) | 45 | 199.7 (6.5) | 8 | <0.001 | 0.4413 |
| MAOB in S100β+ area, a.u. | Fig. 5g | 50 | 110.4 (4.1) | 45 | 140.6(5.2) | 4.5 | <0.001 | 0.1229 |
| ABAT in GFAP+ area, a.u. | Fig. 5i | 50 | 69.0 (3.4) | 38 | 26.1 (1.8) | 8.7 | <0.001 | 0.4837 |
| Putrescine in S100β+ area, a.u. | Fig. S5b | 50 | 3220 (106) | 45 | 4969(146) | 9.8 | <0.001 | 0.4799 |
| S100β+ area, μm^2^. | Fig. 5j | 71 | 448.1 (31.6) | 76 | 740.1 (67.3) | 3.8 | <0.001 | 0.0923 |
| Ramification index-S100β | Fig. 5k | 21 | 1.2 (0.08) | 22 | 1.8 (0.2) | 2.9 | 0.006 | 0.1722 |
| Ending radius-S100β | Fig. 5l | 21 | 67.1(4.0) | 22 | 93.6(5.1) | 4.0 | <0.001 | 0.2870 |
| Sum of intersections-S100β | Fig. 5m | 21 | 12.1(1.0) | 22 | 23.0(2.6) | 3.9 | <0.001 | 0.2655 |
| GFAP+ area, μm^2^ | Fig. 5n | 27 | 235.7(51.6) | 29 | 633.8(113) | 3.1 | 0.003 | 0.1539 |
| Ramification index-GFAP | Fig. S5d | 17 | 1.4 (0.1) | 12 | 2.1 (0.2) | 2.9 | 0.008 | 0.2329 |
| Ending radius-GFAP | Fig. S5e | 17 | 32.7(3.7) | 12 | 61.3(6.3) | 4.0 | <0.001 | 0.3937 |
| Sum of intersections-GFAP | Fig. S5f | 17 | 9.8(1.2) | 12 | 20.2(3.0) | 3.7 | 0.001 | 0.3317 |
| *Electrophysiology* |  |  |  |  |  |  |  |  |
| Tonic GABA current, pA | Fig. 5p | 9 | 2.5(0.7) | 13 | 7.4(1.0) | 3.6 | 0.002 | 0.3974 |
| sIPSC amplitude, pA | Fig. 5q | 9 | 21.4 (1.1) | 13 | 24.0(1.3) | 1.4 | 0.18 | 0.0875 |
| sIPSC frequency, Hz | Fig. 5r | 9 | 2.9(0.4) | 13 | 4.0(0.6) | 1.4 | 0.19 | 0.0840 |
| Spike probability | Fig. 5t | 6 | 0.9(0.08) | 7 | 0.4(0.1) | 3.2 | 0.009 | 0.4739 |
| *Behavioral assessments: fear extinction paradigm* | | |  |  |  |  |  |  |
| Assessment 1 |  |  |  |  |  |  |  |  |
| Retrieval of extinction memory | Fig. S4a | 6 | 17.9(4.4) | 6 | 48.6(6.5) | 3.9 | 0.003 | 0.6051 |
| Retrieval of extinction memory | Fig. 5b | 7 | 7.5(2.0) | 17 | 35.0(5.0) | 3.45 | 0.002 | 0.3508 |
| *Behavioral assessments: working memory* | | | |  |  |  |  |  |
| Y maze spontaneous alternation test |  |  |  |  |  |  |  |  |
| Alternation score | Fig. 5c | 14 | 66.8(2.0) | 14 | 57.4(31) | 2.6 | 0.02 | 0.2042 |
| No. of total entries | Fig. 5d | 14 | 24.3 (2.3) | 14 | 23.6 (2.4) | 0.22 | 0.83 | 0.0440 |
|  | | | | | | | |  |

Table S10. Detailed information about statistical values from PTSD-like mouse model study
The independent t-test was used to compare outcome variables between the Control and PTSD groups. Abbreviations: MAOB, monoamine oxidase B; WT, wild type; KD, knockdown; shRNA, single hairpin RNA; Sc, scramble shRNA; shMAOB, MAOB shRNA; PTSD, posttraumatic stress disorder; SEM, standard error of the mean; GABA, gamma-aminobutyric acid; a.u., arbitrary unit; S100β, S100 calcium-binding protein B; sIPSC, spontaneous inhibitory postsynaptic current.

| Outcome variables | Relevant figure | Sc+PTSD group | | shMAOB+PTSD group | | *t* | *P* | *R^2^* |
| --- | --- | --- | --- | --- | --- | --- | --- | --- |
|  |  | n | mean (SEM) | n | mean (SEM) |  |  |  |
| *Immunohistochemistry* |  |  |  |  |  |  |  |  |
| S100β+ portion in mCherry+ | Fig. 6g | 10 | 88 (2.0) | 10 | 85 (1.9) | 1.06 | 0.31 | 0.0582 |
| GABA in mCherry+ area, a.u. | Fig. 6h | 94 | 149.6 (6.7) | 114 | 69.8 (5.7) | 9.12 | < 0.001 | 0.2878 |
| MAOB in mCherry+ area, a.u. | Fig. 6i | 91 | 80.4 (5.0) | 111 | 36.5 (1.8) | 8.82 | < 0.001 | 0.2800 |
| S100β+ area, μm^2^ | Fig. 6j | 28 | 3,659 (273) | 13 | 1,816 (237) | 4.26 | < 0.001 | 0.3171 |
| Ramification index-S100β | Fig. 6l | 22 | 3.6(0.4) | 22 | 2.5(0.2) | 2.2 | 0.03 | 0.1069 |
| Ending radius-S100β | Fig. 6l | 22 | 74.3(3.9) | 22 | 60.0(4.4) | 2.4 | 0.02 | 0.1317 |
| Sum of intersections-S100β | Fig. 6l | 22 | 25.7(2.2) | 22 | 17.4(1.4) | 3.1 | 0.003 | 0.1983 |
| *Electrophysiology* |  |  |  |  |  |  |  |  |
| Tonic GABA current, pA | Fig. 6o | 7 | 10.67 (1.39) | 6 | 3.33 (0.75) | 4.40 | 0.001 | 0.6535 |
| sIPSC frequency, Hz | Fig. 6p | 7 | 6.10 (0.40) | 6 | 2.83 (0.51) | 5.02 | < 0.001 | 0.0108 |
| sIPSC amplitude, pA | Fig. 6q | 7 | 24.9 (1.6) | 6 | 26.2 (3.6) | 0.35 | 0.74 | 0.5537 |
| *Behavioral assessments: fear extinction paradigm* | | |  |  |  |  |  |  |
| Assessment 1 |  |  |  |  |  |  |  |  |
| Retrieval of extinction memory | Fig. 6c | 15 | 41.4 (4.3) | 15 | 22.8 (3.8) | 3.25 | 0.003 | 0.2733 |
| Retrieval of extinction memory-PL | Fig. S6c | 3 | 26.5(8.5) | 5 | 37.6(11.2) | 0.7 | 0.52 | 0.0726 |
| *Behavioral assessments: working memory and anxiety-like behaviors* | | | |  |  |  |  |  |
| Y maze spontaneous alternation test |  |  |  |  |  |  |  |  |
| Alternation score | Fig. 6d | 9 | 57.5 (3.9) | 10 | 72.5 (2.2) | 3.34 | 0.005 | 0.4089 |
| No. of total entries | Fig. 6e | 9 | 24.3 (2.3) | 10 | 23.6 (2.4) | 0.22 | 0.83 | 0.0028 |
|  | | | | | | | |  |

Table S11. Detailed information about statistical values from genetic mouse model (MAOB-WT vs. MAOB-KD groups).
The independent t-test was used to compare outcome variables between the Sc+PTSD and shMAOB+PTSD groups. Abbreviations: MAOB, monoamine oxidase B; WT, wild type; KD, knockdown; shRNA, single hairpin RNA; Sc, scramble shRNA; shMAOB, MAOB shRNA; PTSD, posttraumatic stress disorder; SEM, standard error of the mean; GABA, gamma-aminobutyric acid; a.u., arbitrary unit; S100β, S100 calcium-binding protein B; sIPSC, spontaneous inhibitory postsynaptic current.

| Outcome variables | Relevant figure | MAOB-WT+PTSD | | MAOB-KO+PTSD | | MAOB-OE+PTSD | | *F* | | *R^2^* | | *P* | | *P* (post-hoc analysis) | | | | |
| --- | --- | --- | --- | --- | --- | --- | --- | --- | --- | --- | --- | --- | --- | --- | --- | --- | --- | --- |
|  |  | n | mean (SEM) | n | mean (SEM) | n | mean (SEM) |  |  |  |  |  |  | MAOB-WT vs.  MAOB-KO | | MAOB-WT vs.  MAOB-OE | MAOB-KO vs.  MAOB-OE | |
| *Immunohistochemistry* |  |  |  |  |  |  |  | |  | |  | |  | |  |  | |  |
| GABA in S100β+ area, a.u. | Fig. 6t | 10 | 266.3 (8.174) | 51 | 154.1(6.856) | 32 | 288.1 (11.01) | 70.55 | | 0.6216 | | <0.001 | | <0.001 | | 0.49 | <0.001 | |
| S100β in S100β+ area, a.u | Fig. 6u | 10 | 3073(105.7) | 51 | 1948(69.3) | 32 | 2608 (150.0) | 19.43 | | 0.3016 | | <0.001 | | <0.001 | | 0.11 | <0.001 | |
| S100β+ area, μm^2^ | Fig. 6v | 10 | 120.4 (15.94) | 51 | 77.22 (5.280) | 32 | 112.6 (11.12) | 6.737 | | 0.1302 | | 0.002 | | 0.03 | | 0.90 | 0.005 | |
| Ramification index-S100β | Fig. 6w | 19 | 2.8 (0.2) | 19 | 2.0(1.6) | 17 | 2.9(0.27) | 5.1 | | 0.1647 | | 0.009 | | 0.3 | | 0.91 | 0.01 | |
| Ending radius-S100β | Fig. 6w | 19 | 22.1 (2.1) | 19 | 11.3 (1.1) | 17 | 29.2 (3.0) | 17.6 | | 0.2110 | | <0.001 | | 0.002 | | 0.06 | <0.001 | |
| Sum of intersections-S100β | Fig. 6w | 19 | 83.7(6.3) | 19 | 57.6(5.5) | 17 | 79.4(4.6) | 6.4 | | 0.3631 | | 0.003 | | 0.004 | | 0.85 | 0.02 | |
| *Fear extinction paradigm* |  |  |  |  |  |  |  |  | |  | |  | |  | |  |  | |
| Retrieval of extinction memory | Fig. 6r | 10 | 30.1 (4.4) | 7 | 14.7 (3.4) | 5 | 36.3 (5.1) | 5.5 | | 0.3666 | | 0.01 | | 0.04 | | 0.62 | 0.02 | |
|  | | | | | | | | | | | | | | | | | | |

**Table S12. Detailed information about statistical values from genetic mouse model (MAOB-WT vs. MAOB-KO vs. MAOB-OE)**
One-way analysis of variance (ANOVA) was used to compare outcome variables among the WT+PTSD, MAOB-KO+PTSD, and MAOB-OE+PTSD groups. Tukey multiple comparison tests were applied for pairwise comparisons of group means.

Abbreviations: MAOB, monoamine oxidase B; WT, wild type; KO, knockout; PTSD, posttraumatic stress disorder; OE, overexpression; SEM, standard error of the mean.

| Outcome variables | Relevant figure | Control | | PTSD | | PTSD+KDS2010 | | Statistical values* | | *R^2^* | *P* | | *P* (post-hoc analysis) | | | | |
| --- | --- | --- | --- | --- | --- | --- | --- | --- | --- | --- | --- | --- | --- | --- | --- | --- | --- |
|  |  | n | mean (SEM) | n | mean (SEM) | n | mean (SEM) |  |  |  |  |  | Control vs. PTSD | | Control vs. PTSD+KDS | | PTSD vs.  PTSD+KDS |
| *Immunohistochemistry* |  |  |  |  |  |  |  | |  | |  |  | |  | |  | |
| GABA in S100β+ area, a.u. | Fig. 7f | 119 | 92.7 (6.1) | 134 | 164.2 (5.4) | 106 | 99.7 (6.3) | 46.9 | | 0.1136 | < 0.001 | | < 0.001 | | 0.70 | | < 0.001 |
| GABA in NeuN+ area, a.u. | Fig. S6b | 270 | 7307 (109.6) | 309 | 7856 (172.5) | 210 | 7843 (213.2) | 3.63 | | 0.0032 | 0.03 | | 0.04 | | 0.08 | | >0.99 |
| MAOB in S100β+ area, a.u. | Fig. 7g | 119 | 85.1 (5.5) | 134 | 146.9 (5.2) | 26 | 106.0 (3.8) | 65.7 | | 0.2697 | < 0.001 | | < 0.001 | | 0.23 | | < 0.001 |
| Putrescine in S100β+ area, a.u. | Fig. S6f | 119 | 2709 (73.7) | 50 | 4981 (158.0) | 85 | 4581 (116.3) | 141.0 | | 0.5292 | < 0.001 | | < 0.001 | | < 0.001 | | 0.05 |
| ABAT in GFAP+ area, a.u. | Fig. 7i | 55 | 168.5 (6.5) | 44 | 146.9 (6.6) | 41 | 168.8 (6.2) | 3.63 | | 0.0574 | 0.03 | | 0.04 | | 0.95 | | 0.03 |
| S100β+ area, μm^2^ | Fig. 7j | 119 | 407.0 (28.5) | 134 | 624.9 (45.6) | 106 | 487.6 (34.7) | 8.9 | | 0.0474 | < 0.001 | | < 0.001 | | 0.32 | | 0.03 |
| GFAP+ area, pixel^2^ | Fig. 7k | 25 | 117.7 (12.1) | 36 | 191.9 (9.9) | 26 | 119.2 (13.8) | 14.29 | | 0.2539 | < 0.001 | | < 0.001 | | > 0.99 | | < 0.001 |
| Ramification index | Fig. 7l | 21 | 1.2 (0.08) | 22 | 1.8 (0.17) | 16 | 1.3 (0.08) | 6.49 | | 0.1881 | 0.003 | | 0.006 | | 0.99 | | 0.02 |
| Ending radius | Fig. 7m | 21 | 67.14 (4.0) | 22 | 93.6 (5.1) | 16 | 56.9 (3.4) | 18.46 | | 0.3973 | < 0.001 | | < 0.001 | | 0.26 | | < 0.001 |
| Sum of intersections | Fig. 7n | 21 | 12.1 (1.04) | 22 | 23.0 (2.6) | 16 | 9.7 (0.8) | 15.31 | | 0.3534 | < 0.001 | | < 0.001 | | 0.66 | | < 0.001 |
| NeuN+ area, μm^2^ | Fig. S6c | 270 | 415.0 (17.6) | 308 | 465.2 (27.1) | 210 | 450.6 (22.5) | 1.29 | | 0.0032 | 0.28 | | 0.25 | | 0.57 | | 0.9 |
| Number of NeuN | Fig. S7d | 7 | 33.6 (3.2) | 7 | 32.8 (3.9) | 8 | 31.0 (2.4) | 0.19 | | 0.0200 | 0.83 | | 0.98 | | 0.82 | | 0.91 |
| *Electrophysiology* |  |  |  |  |  |  |  |  | |  |  | |  | |  | |  |
| Tonic GABA current, pA | Fig. 7p | 9 | 2.52 (0.69) | 13 | 7.44 (1.01) | 7 | 2.54 (0.64) | 10.57 | | 0.4485 | < 0.001 | | 0.001 | | > 0.99 | | 0.003 |
| sIPSC amplitude, pA | Fig. 7q | 9 | 21.0 (1.3) | 13 | 24.9 (1.5) | 7 | 24.5 (1.9) | 1.77 | | 0.1199 | 0.19 | | 0.19 | | 0.36 | | 0.98 |
| sIPSC frequency, Hz | Fig. 7r | 9 | 2.89 (0.40) | 13 | 4.41 (0.71) | 7 | 3.05 (0.55) | 1.88 | | 0.1265 | 0.17 | | 0.21 | | 0.99 | | 0.33 |
| Spike probability at 300 μA | Fig. 4I | 6 | 1.0 (0) | 7 | 0.47 (0.16) | 7 | 0.9 (0.04) | 7.53 | | 0.4697 | 0.005 | | 0.006 | | 0.78 | | 0.02 |
| *CBF measurements* |  |  |  |  |  |  |  |  | |  |  | |  | |  | |  |
| Peak amplitude, % | Fig. 7w | 28 | 17.7 (6.6) | 43 | 13.2 (5.4) | 50 | 19.5 (9.4) | 8.33 | | 0.1237 | < 0.001 | | 0.007 | | > 0.99 | | < 0.001 |
| AUC, a.u. | Fig. 7x | 28 | 8,164 (3,777) | 43 | 6,058 (3,434) | 50 | 10,049 (5,769) | 8.65 | | 0.1279 | < 0.001 | | 0.03 | | 0.37 | | < 0.001 |
| Time to peak, sec | Fig. 7y | 28 | 50.5 (17.5) | 43 | 53.6 (23.9) | 50 | 53.2 (16.4) | 0.23 | | 0.0039 | 0.79 | | 0.79 | | 0.84 | | > 0.99 |
| *Behavioral assessments: fear extinction paradigm* | | | |  |  |  |  |  | |  |  | |  | |  | |  |
| Assessment 1 |  |  |  |  |  |  |  |  | |  |  | |  | |  | |  |
| Retrieval of extinction | Fig. 7b |  | NA | 17 | 35.0 (5.0) | 15 | 16.9 (3.9) | 2.80 | | 0.4222 | 0.009 | | NA | | NA | | NA |
| *Behavioral assessments: working memory* | | | | | |  |  |  | |  |  | |  | |  | |  |
| Y maze spontaneous alternation test | |  |  |  |  |  |  |  | |  |  | |  | |  | |  |
| Alternation score | Fig. 7c | 27 | 66.1 (1.3) | 27 | 56.8 (1.9) | 11 | 64.6 (1.2) | 10.3 | | 0.2486 | < 0.001 | | < 0.001 | | 0.86 | | 0.02 |
| No. of total entries | Fig. 7d | 27 | 26.6 (1.4) | 27 | 21.5 (1.3) | 11 | 32.6 (2.3) | 10.4 | | 0.1350 | < 0.001 | | 0.03 | | 0.05 | | < 0.001 |
|  | | | | | | | | | | | | | | | | | |

Table S13. Detailed information about the statistical values from MAOB-inhibitor drug therapy
One-way analysis of variance (ANOVA) was used to compare outcome variables among the control, PTSD, and PTSD+KDS2010 mouse groups. Tukey multiple comparisons tests were applied for pairwise comparisons of group means. Comparisons of outcome measures from behavioral assessments 1 and 2 were performed between the PTSD and PTSD+KDS2010 mouse groups using the independent t-test or Mann-Whitney U test, as appropriate. * F value for the one-way ANOVA, t value for the independent t-test, or U value for the Mann-Whitney U test. Abbreviations: PTSD, posttraumatic stress disorder; SEM, standard error of the mean; GABA, gamma-aminobutyric acid; S100β, S100 calcium-binding protein B; a.u., arbitrary unit; MAOB, monoamine oxidase B; GFAP, glial fibrillary acidic protein; ABAT, 4-aminobutyrate aminotransferase; sIPSC, spontaneous inhibitory postsynaptic current; AUC, area under the curve; NA, not available.
