# Supplementary material for: Astrocytic gamma-aminobutyric acid dysregulation as a therapeutic target for posttraumatic stress disorder
Source: Signal Transduct Target Ther. 2025 Jul 28;10:240. doi: 10.1038/s41392-025-02317-5 (PMC12301456; doi:10.1038/s41392-025-02317-5)
Supplement: Supplementary file 2 — Clinical study protocol [file 41392_2025_2317_MOESM2_ESM.pdf]

Study Protocol:
The effects of trauma exposure on the brain, behavior, and cognition: A longitudinal neuroimaging study

1. BACKGROUND

Posttraumatic stress disorder (PTSD) is a psychiatric condition that can develop following exposure to traumatic events involving a serious threat to life, physical injury, or witnessing the death or suffering of others. Despite the widespread occurrence of trauma in the general population, only a subset of individuals develop PTSD, indicating that individual differences in neurobiological sensitivity and psychological resilience play crucial roles in determining clinical outcomes. Identifying the mechanisms underlying this variability remains a fundamental question in psychiatric neuroscience.

Previous neuroimaging studies have revealed alterations in brain networks involved in emotional regulation, threat detection, and stress reactivity in individuals with PTSD. These studies suggest that PTSD is associated with both functional and structural brain changes. However, most of this work has relied on cross-sectional data, making it difficult to determine whether the observed neural abnormalities precede the development of symptoms, result from trauma exposure, or persist as enduring traits in chronic cases. Without longitudinal evidence, it remains unclear whether these neural signatures represent markers of vulnerability, illness maintenance, or recovery.

The need for longitudinal research is particularly urgent in civilian trauma populations, which have received comparatively less attention than military or disaster-exposed cohorts. In everyday life, individuals are more likely to experience trauma through interpersonal violence, serious accidents, or medical crises rather than through war or large-scale disasters. Nevertheless, most existing PTSD studies have focused on highly specific groups, limiting their applicability to broader civilian populations. Furthermore, developmental aspects of trauma responses remain underexplored. Adolescents and young adults, whose brains are still undergoing rapid maturation, may be particularly vulnerable to long-term neurobiological consequences of trauma exposure, or alternatively, may exhibit unique patterns of resilience.

Emerging evidence, including our own prior work, suggests that changes in neurochemical signaling and cerebral blood flow within stress-regulatory brain regions may contribute to differences in clinical trajectories following trauma. Such changes may serve as potential biomarkers for identifying individuals at risk for chronic PTSD or those likely to experience spontaneous recovery. However, these findings require validation in large, systematically studied civilian samples using multimodal brain imaging and repeated longitudinal assessments over time.

2. OBJECTIVES OF STUDY

This naturalistic observational study aims to investigate the biological and psychological mechanisms underlying the development and recovery of PTSD in a civilian sample following exposure to various traumatic events. Through comprehensive longitudinal assessments of brain structure and function, behavior, and cognition in trauma-exposed individuals, the study seeks to identify key factors contributing to individual differences in clinical outcomes. Specifically, the study will examine neuroimaging characteristics, psychosocial influences, peripheral blood-based biomarkers, and genetic predispositions that are associated with the onset, persistence, or remission of PTSD.

The ultimate goal of this study is to elucidate the pathophysiological mechanisms of PTSD and to discover predictive and protective factors that may inform the development of early diagnostic tools and more effective, biologically grounded treatments.

3. STUDY DESIGN

This study is a naturalistic observational study involving adults and adolescents with and without trauma exposure. Participants will be recruited into trauma-exposed and trauma-unexposed groups.

At baseline, all participants will undergo comprehensive multi-modal neuroimaging, neurophysiological, clinical, and cognitive assessments. Follow-up evaluations will be conducted flexibly, adapting to the natural course of participant engagement and clinical needs. While we aim for periodic assessments, including potential evaluations around 3 months, 6 months, 1 year,

2 years, within 5 years, and within 10 years, the precise timing and frequency of these follow-ups will depend on individual participant availability and consent, reflecting the real-world nature of this study. The same protocols as baseline will be utilized for all follow-up assessments to ensure consistency.

4. STUDY POPULATION

4.1. Participant Eligibility Criteria

To participate in this study, individuals must meet the following criteria:

(1) Age: Male and female participants aged 12 to 65 years old

(2) Consent: Provided written consent to participate in the study

(3) Trauma exposure:

- Trauma-exposed group: Individuals who have experienced traumatic events, defined according to the criteria for Criterion A (Exposure to a Traumatic Event) in the Diagnostic and Statistical Manual of Mental Disorders (DSM)
- Trauma-unexposed group (healthy control group): Individuals who have not experienced traumatic events

4.2. Exclusion Criteria

Individuals will be excluded from the study if they meet any of following criteria:

(1) For all participants: Presence of significant or unstable medical conditions necessitating hospitalization or surgical intervention

(2) For the Trauma-unexposed group only:

- Diagnosis of any psychiatric disorder
- History of at least one major depressive episode within the past 12 months prior to enrollment
- Current diagnosis of alcohol or other substance dependence

(3) For participants undergoing neuroimaging assessments only:

- Pregnancy or lactation

- History of neurological abnormalities or physical conditions known to affect brain imaging data, such as traumatic brain injury, epilepsy, multiple sclerosis, brain tumors, or cerebrovascular disease
- Any contraindications to magnetic resonance imaging (MRI) procedures, including severe claustrophobia or the presence of metal implants
- Use of psychotropic medications which can affect neuroimaging within the past three months (specific to trauma-unexposed participants)

4.3. Withdrawal Criteria

Participant will be withdrawn from the study if he/she meets any of the following conditions:

(1) Violation of the inclusion criteria: The participant is found to have violated any of pre-defined inclusion criteria.

(2) Participant request: The participant requests to withdraw from the study.

(3) Fulfillment of exclusion criteria: The participant subsequently meets any of the exclusion criteria (e.g., development of a new, severe, major physical illness).

(4) Investigator discretion for safety: The investigator deems it necessary for the participants' safety.

(5) Significant non-compliance: The participant demonstrates a significant degree of non-compliance with study procedures.

(6) Loss to follow-up: The participant is lost to contact, making longitudinal follow-up impossible.

5. ASSESSMENTS

The following assessments will be conducted at both baseline and follow-up, as feasible and subject to participant consent and willingness to participate in specific evaluations:

(1) Demographic and clinical data collection: This includes a comprehensive collection of demographic and clinical data (e.g., age, sex, education, socioeconomic status, marital status, handedness, etc.), a physical examination, and standard laboratory testing (complete blood count and blood chemistry).

(2) Traumatic event assessment: We will assess the types and timing of traumatic events experienced across the life span, using both quantitative and qualitative evaluation methods. This includes, but is not limited to, characterizing events by their nature (e.g., single-incident vs. repeated/chronic exposure), presence of physical injury, and age of first exposure.

(3) Psychiatric evaluation: This involves a comprehensive evaluation of the participant's mental status, including PTSD, using standardized clinical interviews as Structured Clinical Interview for DSM (SCID) and the Clinician-Administered PTSD Scale for DSM (CAPS). Additionally, a battery of validated questionnaire will be utilized to measure various aspects of psychopathology, including depression, anxiety, alcohol use, smoking habit, suicide, resilience, anger, and impulsivity.

(4) Multimodal brain imaging: This may include high-resolution T1-weighted imaging, proton magnetic resonance spectroscopy (1H-MRS), arterial spin labeling (ASL), resting-state functional MRI, and diffusion tensor imaging (DTI), which will be obtained using 3.0 Tesla MRI scanners. The specific imaging modalities used will depend on participant consent and feasibility of each scan.

(5) Neurophysiological testing: This includes skin conductance response, and heart rate variability

(6) Comprehensive neuropsychological assessment: A comprehensive neuropsychological assessment covering various cognitive domains will be conducted.

(7) Peripheral blood biomarker analysis and genetic/epigenetic profiling

6. STATISTICAL ANALYSIS PLAN

Statistical analysis will be conducted using a rigorous approach to examine both cross-sectional differences and longitudinal changes, as well as to identify associations between various measures.

6.1. General Principles and Methods

(1) Cross-sectional between-group comparisons: For continuous demographic variables, one-way ANOVA or t-test will be employed. Categorical variables will be compared using Fisher's exact test. For other cross-sectional comparisons of neuroimaging, clinical,

neurophysiological, and neuropsychological characteristics between groups, independent t-tests, chi-squared tests, or analysis of covariance (ANCOVA), or multiple linear regression analysis will be utilized, as appropriates. Potential covariates such as age, sex, and other relevant factors, will be considered, as necessary.

(2) Longitudinal Analyses: To assess longitudinal changes in neuroimaging, clinical, neurophysiological, and neuropsychological measures, repeated measures ANCOVA or linear mixed-effects models will be employed. These models are robust for handling within-subject correlations and missing data inherent in longitudinal designs.

(3) Assessment of Associations: Correlations between clinical severity, scale scores, and other assessment findings will be examined using Pearson's correlation or Spearman's correlation. To control for potential confounding variables and assess independent relationships, multiple linear regression or linear mixed-effects models will be used, as appropriate.

(4) Neuroimaging-Specific Analyses:

- Structural and Functional Brain Changes: We will compare and analyze structural and functional brain changes between groups.
- MRS Neurometabolite Analysis: For the analysis of individual MRS neurometabolites, the LCModel or appropriate post-processing tools will be used.
- Graphical Analysis and Presentation: For graphical analysis and presentation of brain imaging data, SPM (Statistical Parametric Mapping) and FSL (FMRIB Software Library), or other neuroimaging analysis softwares will be utilized.

6.2. Statistical Significance and Multiple Comparisons:

Statistical significance will be defined as a two-tailed p-value < 0.05. For general statistical analyses, Bonferroni correction or other correction methods will be applied to address multiple comparisons where appropriate. For neuroimaging analyses, more specialized methods for multiple comparison correction will be considered, including Family-Wise Error (FWE) rate, False Discovery Rate (FDR), permutation testing, or Monte Carlo simulation, with significance also defined at a p-value < 0.05. The specific correction method will be chosen based on the nature of the analysis and data characteristics
